# Regulation of cancer-associated fibroblasts for enhanced cancer immunotherapy using advanced functional nanomedicines: an updated review

**DOI:** 10.1186/s12951-025-03217-0

**Published:** 2025-03-04

**Authors:** Tingting Liao, Xiaoxiao Chen, Fengkai Qiu, Xinyu Zhang, Fazong Wu, Zhongwei Zhao, Ming Xu, Minjiang Chen, Jia-Wei Shen, Qiying Shen, Jiansong Ji

**Affiliations:** 1 School of Pharmacy, College of Pharmacy, Hangzhou Normal University, 2318 Yuhangtang Road, Hangzhou, 310015 Zhejiang China; 2 Zhejiang Key Laboratory of Imaging and Interventional Medicine, The Fifth Affiliated Hospital of Wenzhou Medical University, 289 Kuocang Road, Lishui, 323000 China; 3 Department of Radiology, Lishui Central Hospital, The Fifth Affiliated Hospital of Wenzhou Medical University, Lishui, 323000 China; 4 Cixi Biomedical Research Institute, Wenzhou Medical University, Ningbo, 315300 China; 5 Key Laboratory of Elemene Class Anti-Cancer Chinese Medicines, Engineering Laboratory of Development and Application of Traditional Chinese Medicines, Collaborative Innovation Center of Traditional Chinese Medicines of Zhejiang Province, Hangzhou Normal University, Hangzhou, 311121 China

**Keywords:** Cancer-associated fibroblasts (CAFs), Tumor microenvironment, Immunosuppression, Immunotherapy enhancement, Nano-delivery systems

## Abstract

The tumor microenvironment (TME) is a complex and dynamic ecosystem that plays a critical role in cancer progression. It comprises various cell types, including immune cells, tumor cells, and stromal cells. Among these, cancer-associated fibroblasts (CAFs) represent a heterogeneous population with diverse origins, phenotypes, and functions. Activated CAFs secrete multiple factors that promote tumor growth, migration, angiogenesis, and contribute to chemoresistance. Additionally, CAFs secrete extracellular matrix (ECM) components, such as collagen, which form a physical barrier that hinders the penetration of chemotherapeutic and immunotherapeutic agents. This ECM also influences immune cell infiltration, impeding their ability to effectively target tumor cells. As a result, modulating the activity of CAFs has emerged as a promising strategy to enhance the efficacy of tumor immunotherapy. Nano-delivery systems, constructed from various nanomaterials with high targeting specificity and biocompatibility, offer a compelling approach to deliver therapeutic agents or immunomodulatory factors directly to CAFs. This modulation can alter CAF function, reduce their tumor-promoting effects, and thereby improve the outcomes of immunotherapy. This review provides an in-depth exploration of the origins, functions, and interactions of CAFs within the TME, particularly in the context of immune suppression. Furthermore, it discusses the potential applications of functional nanocarrifers in modulating CAFs and enhancing the effectiveness of tumor immunotherapy, highlighting the significant progress and potential of nanotechnology in this area.

**Graphical Abstract:**

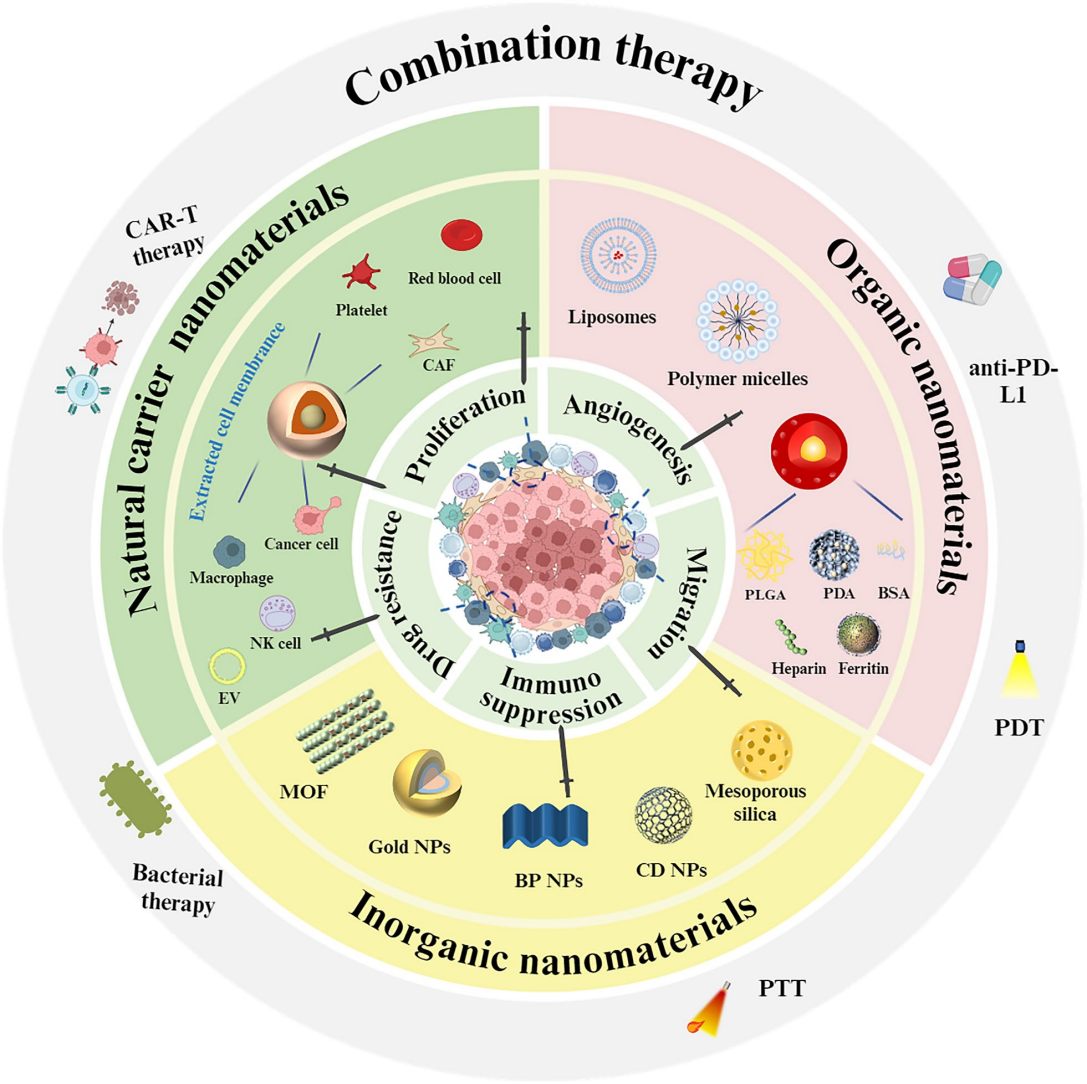

## Introduction

The global burden of cancer continues to rise, posing a formidable public health challenge worldwide. Despite significant advancements in the understanding of cancer biology, it is increasingly clear that cancer is not solely the result of genetic mutations in tumor cells, but rather arises within a complex and dynamic tumor microenvironment (TME) [1]. The TME comprises a heterogeneous mixture of cellular and non-cellular components, including tumor cells, immune cells, cancer-associated fibroblasts (CAFs), extracellular matrix (ECM) elements, and soluble factors such as growth factors and cytokines [2]. These components engage in intricate and reciprocal interactions that play crucial roles in regulating tumor growth, metastasis, and the tumor’s response to therapeutic interventions.

Among the key cellular players within the TME, CAFs are a highly heterogeneous population of stromal cells that significantly contribute to tumor progression. CAFs are derived from normal fibroblasts but undergo phenotypic and functional transformations in response to tumor-derived signals, including cytokines, growth factors, and ECM remodeling enzymes [3]. These activated fibroblasts reside within the TME, where they facilitate tumor progression through multiple mechanisms. CAFs promote tumor growth and metastasis by secreting immune-suppressive factors (e.g., IL-6, TGF-β, CXCL12) and by altering the physical properties of the tumor stroma [4–6]. They also interact with various immune cells, including macrophages, myeloid-derived suppressor cells (MDSCs), and regulatory T cells (Tregs), thereby contributing to an immunosuppressive microenvironment [3, 6]. These interactions hinder the activity of effector T cells, facilitating the tumor’s evasion of host immune surveillance [7, 8]. Given their pivotal role in shaping the immunosuppressive TME, CAFs have emerged as an important therapeutic target in cancer immunotherapy.

Current strategies to modulate CAFs primarily focus on both direct and indirect approaches to alter their activation, function, and interactions with the TME [9–11]. These approaches include targeting key signaling pathways such as TGF-β [12, 13], Hedgehog [14], and Wnt/β-catenin [15], which are known to regulate CAF activation and their subsequent contributions to tumor progression. Additionally, immunomodulatory strategies—such as modulation of immune checkpoint molecules and the use of CAF-targeting antibodies—aim to enhance anti-tumor immunity by disrupting the immunosuppressive microenvironment created by CAFs [16–19]. Moreover, the application of nanomaterials offers significant promise in enhancing the solubility, stability, and sustained release of therapeutic agents, while also enabling targeted delivery to CAFs and other components of the TME [20–22]. Nanomaterial-based drug delivery systems hold the potential to selectively target CAFs, modulate their activity within the TME, improve drug penetration, facilitate immune cell infiltration, and counteract the immunosuppressive effects exerted by CAFs [21, 23–25]. Although targeting CAFs has demonstrated promise in tumor therapy, several limitations persist, particularly the heterogeneity of CAFs and the complexity of the TME [26]. These challenges highlight the necessity for a more comprehensive understanding of the intricate interactions between CAFs and their surrounding environment. A deeper elucidation of these interactions is essential for the development of more effective and targeted therapeutic strategies.

In this review, we explore the complex interactions between CAFs and immune cells within the TME, with a focus on the role of CAFs in shaping the tumor immune microenvironment through various metabolic and signaling pathways. We also discuss the potential of functional nanocarriers to modulate CAF activity, enhance anti-tumor immune responses, and optimize cancer treatment outcomes by overcoming the structural and immunosuppressive barriers imposed by the TME. Strategies aimed at inhibiting CAF activation, interfering with CAF functions, and selectively depleting or killing CAFs are examined in detail. However, despite the promising therapeutic potential, these approaches face several challenges that need to be addressed to improve their clinical efficacy.

## CAFs’ origin and heterogeneity

**Fig. 1:**
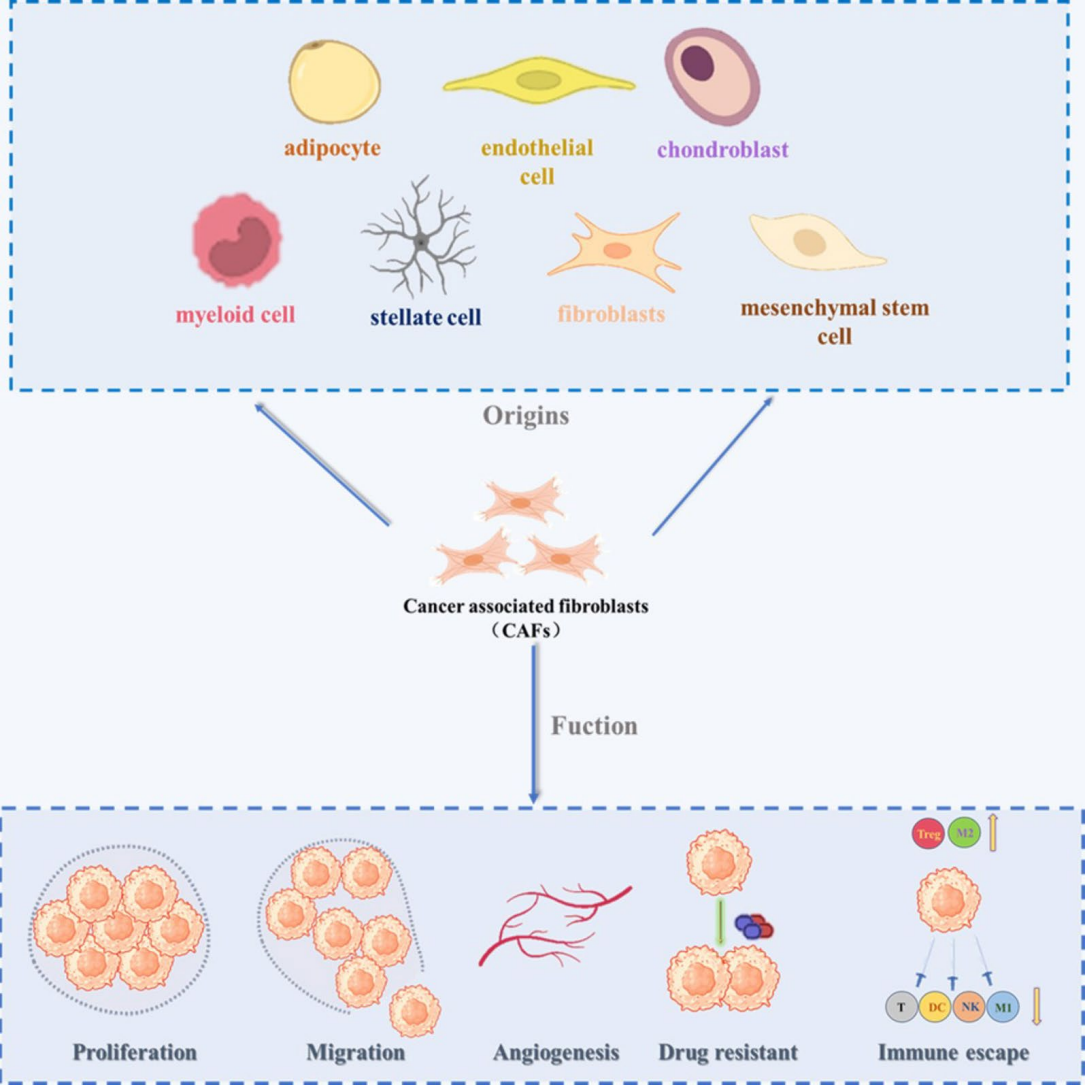
The origins of CAFs and their multifaceted roles in tumor development and therapy. CAFs originate from various cell types, including adipocytes, endothelial cells, chondrocytes, myoblast cells, stellate cells, fibroblasts, and mesenchymal stem cells. Upon activation by tumor-derived signals, CAFs undergo phenotypic and functional changes that enable them to contribute to various aspects of tumor progression

CAFs are a diverse and dynamic population of cells that contribute significantly to the TME. The origin and heterogeneity of CAFs have been extensively studied, as these cells play key roles in tumor progression, metastasis, and therapy resistance (Fig. 1). CAFs are derived from multiple sources, including resident fibroblasts, endothelial cells, perivascular cells, and bone marrow-derived mesenchymal stem cells MSCs [3]. Upon exposure to tumor-derived signals such as growth factors (e.g., TGF-β, PDGF) and cytokines (e.g., IL-6), these precursor cells undergo activation and reprogramming, acquiring a myofibroblast-like phenotype characterized by the expression of markers such as α-smooth muscle actin (α-SMA) and fibroblast activation protein (FAP) [5, 27, 28].

The heterogeneity of CAFs is a defining feature that complicates their study and therapeutic targeting. CAFs are not a uniform population, but rather consist of distinct subtypes with diverse molecular profiles and functional roles. Recent research has identified at least two major subtypes of CAFs based on their molecular signatures: the myofibroblastic CAFs (myCAFs), which are involved in ECM remodeling and tissue stiffness, and the inflammatory CAFs (iCAFs), which produce pro-inflammatory cytokines such as IL-6 and IL-8, contributing to immune modulation and tumor progression [29–31]. Additionally, antigen-presenting CAFs (apCAFs) have been shown to influence immune cell infiltration and anti-tumor immunity [32]. These subpopulations of CAFs exhibit different capacities to modulate tumor behavior, with some facilitating tumor growth and metastasis, while others may suppress certain aspects of malignancy. The functional diversity and plasticity of CAFs are shaped by a combination of intrinsic factors, such as genetic mutations, and extrinsic signals from the TME, including hypoxia, altered metabolism, and tumor-derived exosomes [33]. This remarkable adaptability allows CAFs to support various stages of tumor progression, including EMT, angiogenesis, and immune evasion [34].

Overall, the origin and heterogeneity of CAFs are fundamental to their diverse roles in cancer biology. The identification of distinct CAF subpopulations and their specific contributions to the TME underscores the complexity of their functions. A better understanding of the molecular mechanisms driving CAF differentiation and plasticity will be crucial for developing targeted therapies aimed at modulating CAF activity in cancer treatment [2].

## Role of CAFs in the TME

**Fig. 2:**
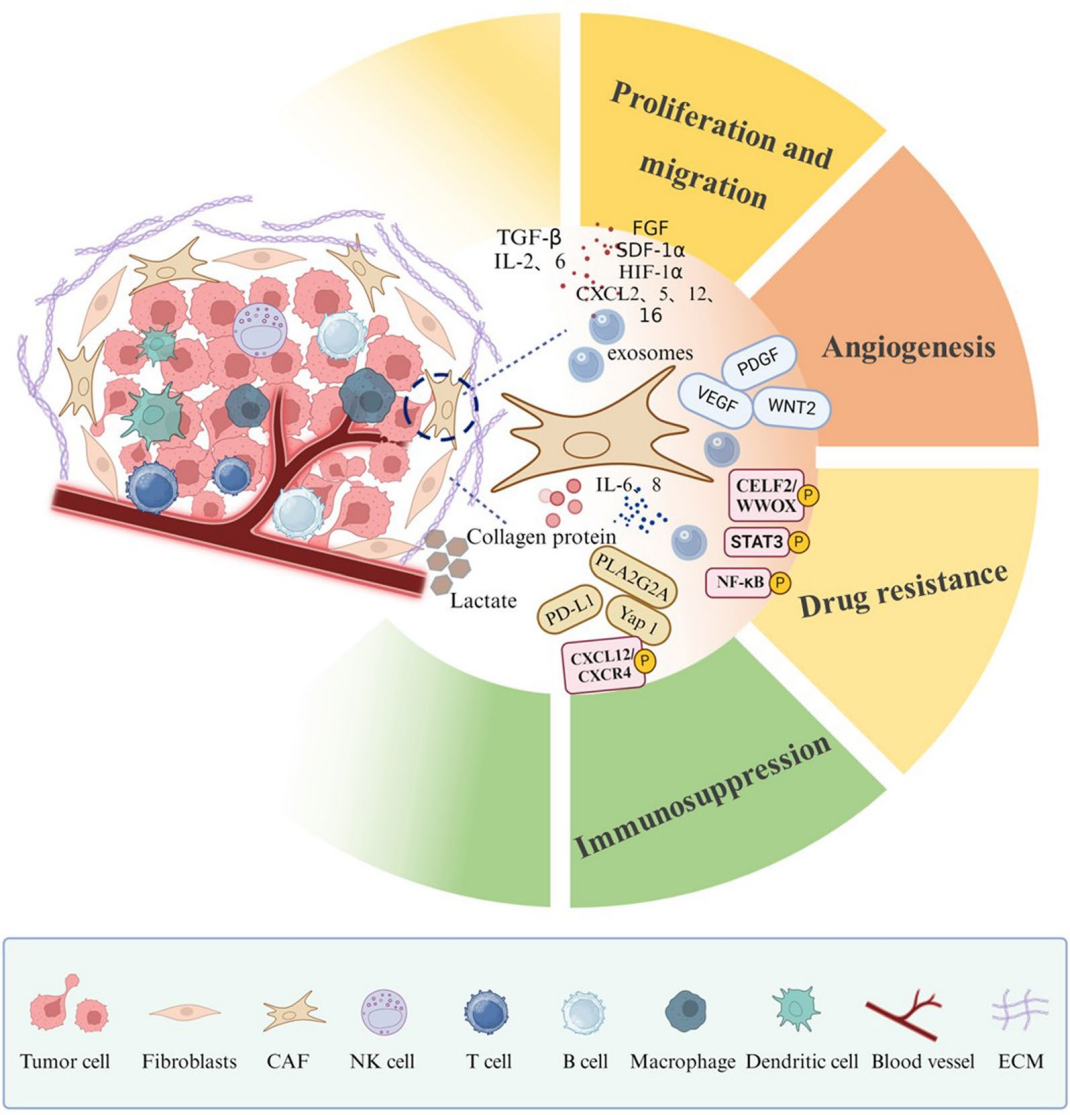
The Role of CAFs in the TME.CAFs support tumor growth, migration, angiogenesis, drug resistance, and immune suppression by secreting factors like TGF-β, IL-6, VEGF, and PDGF. They enhance angiogenesis, promote drug resistance via STAT3 and NF-κB, and recruit immune cells such as Tregs and MDSCs. CAFs also remodel the extracellular matrix, affecting immune cell infiltration, while CAF-derived exosomes further impact tumor progression and immunity

CAFs secrete factors like TGF-β, IL-6, VEGF, and PDGF, which drive tumor progression by enhancing angiogenesis and creating a pro-tumorigenic microenvironment that supports tumor cell survival and migration [34]. CAFs also play a critical role in therapeutic resistance, activating signaling pathways such as STAT3 and NF-κB, which help tumor cells evade chemotherapy and radiotherapy [35, 36]. Furthermore, CAFs influence immune responses by recruiting and activating immune suppressive cells, including Tregs and MDSCs, thereby impairing the efficacy of immunotherapies [6]. They also remodel the extracellular matrix, promoting tumor invasion and further inhibiting immune cell infiltration, which contributes to immune evasion [3]. CAF-derived exosomes, which carry bioactive molecules, facilitate communication between CAFs and other TME cells, enhancing tumor progression and metastasis [37, 38]. Thus, CAFs play a multifaceted role in shaping the TME, promoting tumor growth, and limiting the effectiveness of both immune and conventional therapies. Given the substantial impact of CAFs on tumor biology, their role in the TME represents an area of intense research, with ongoing efforts aimed at understanding their functional diversity and developing therapeutic approaches to modulate their activity in the context of cancer (Fig. 2).

### Promote tumor proliferation and migration

One of the major components of the TME, CAFs contribute significantly to tumor progression by promoting cell proliferation, migration, and metastasis. This is primarily achieved through the secretion of various bioactive factors, including transforming growth factor-beta (TGF-β), interleukins (IL-2, IL-6), C-X-C motif chemokine ligands (CXCL2, CXCL5, CXCL12, CXCL16), fibroblast growth factor 7 (FGF7), and hypoxia-inducible factor (HIF1A/HIF-1α) [3].For example, TGF-β induces CAF activation, thereby promoting tumor fibroplasia and facilitating tumor progression through its autocrine and paracrine effects. Studies have implicated the TGF-β pathway in the promotion of prostate cancer cell proliferation and migration [39], while overexpression of TGF-β1 and SDF-1α in cervical cancer enhances cell growth and invasiveness [40]. CAFs also modulate the Wnt/β-catenin and TGF-β/SMAD pathways, influencing AKT and MAPK signaling through the secretion of VCAM1, thereby impacting tumor growth and invasion [41, 42].Under hypoxic conditions, CAFs regulate HIF-1α-dependent glycolytic signaling, affecting ovarian cancer cell invasion and migration by modulating CRMP2 [43]. Additionally, FGF7 secreted by CAFs influences HIF-1α signaling and epithelial-mesenchymal transition EMT [44]. CAFs contribute to ECM production by secreting CXCL16 and enhancing MMP expression, which increases tumor stiffness and alters ECM structure [45].The binding of PF4/CXCL4 and PPBP/CXCL7 to CXCL12, mediated by G protein-coupled receptors, promotes EMT and the expression of chemokines and cytokines [46]. In hepatocellular carcinoma (HCC), versican (VCAN) secreted by CAFs is associated with malignant transformation and prognosis [47]. SERPINE1/PAI1 from CAFs induces the transformation of lymphatic endothelial cells into mesenchymal cells in cervical squamous cell carcinoma, facilitating cancer metastasis [48].Furthermore, CAFs secrete exosomes that actively participate in cancer cell proliferation and invasion. These exosomes contain specific miRNAs, such as miR-20a-5p and miR-421, which promote IL-6 production and pancrea1ic cancer progression, respectively, by regulating the SIRT3/H3K9ac/HIF-1α axis [37, 49].The cargo of CAF-derived exosomes is crucial for their effectiveness in cancer progression.

In summary, CAFs play multifaceted roles in promoting tumor growth, invasion, and metastasis through complex signaling networks, ECM remodeling, and exosome-mediated communication, making them critical players in cancer biology and potential targets for therapeutic interventions.

### Promote tumor angiogenesis

CAFs possess the ability to secrete a variety of pro-angiogenic factors, including vascular endothelial growth factors (VEGFs), PDGFs, and Wnt family member 2 (WNT2). These factors promote endothelial cell proliferation by binding to their corresponding receptors and activating the corresponding signaling pathways, which in turn promote angiogenesis and tumor development in vivo [50]. For example, VEGF, a potent angiogenic and vascular permeability factor, can significantly affect the ECM when secreted by CAFs, promoting an increase in vascular volume and permeability, which in turn contributes to enhanced tumor metastasis [51]. In addition, PDGFs secreted by CAFs can further promote angiogenesis by influencing matrix activation [52]. In CRC, reducing WNT2 expression in CAFs significantly reduced angiogenesis, whereas overexpressing WNT2 increased vessel density and tumor volume [53]. Moreover, CAFs affect MMP degradation function by downregulating SRY-box transcription factor 4 (SOX4), further affecting angiogenesis and tumor metastasis [54]. Furthermore, CAFs can infiltrate the TME by attracting immune cells such as TAMs [55]. For example, in head and neck squamous cell carcinoma, CAFs regulate the YAP1/HIF-1α axis by delivering miR-21-5p to endothelial cells, thereby promoting tumor angiogenesis [38]

### Increasing tumor drug resistance

CAFs contribute to tumor drug resistance through complex mechanisms, primarily by modulating key signaling pathways. One such pathway is the signal transducer and activator of transcription 3 (STAT3), which is often hyperactivated in cancer. This can occur via cytokine signaling (e.g., IL-6) or interactions with other pathways like nuclear factor kappa B (NF-κB) [56]. CAFs secrete IL-6, which activates STAT3 and reduces the efficacy of chemotherapies like gemcitabine [35]. Additionally, CAF-derived exosomal miR-21 enhances STAT3 activation and induces MDSC production [57], promoting cisplatin resistance in esophageal squamous cell carcinoma [58]. CAFs also influence tumor drug resistance through exosome-mediated signaling. In colorectal cancer (CRC), CAF-secreted miR-625-3p via exosomes enhances the CELF2/WWOX pathway, promoting tumor growth and chemoresistance [59]. In pancreatic cancer, CAFs release circFARP1, which activates STAT3 and augments resistance to gemcitabine [60]. Conversely, circZFR in HCC inhibits the STAT3/NF-κB pathway, promoting cisplatin resistance. CAFs further contribute to tumor resistance by secreting extracellular matrix (ECM components like HA and collagenase), which alters drug diffusion within the TME [61]. Metabolic reprogramming also plays a role, as lactic acid secreted by cancer cells transforms stromal fibroblasts into CAFs [36]. This transformation, coupled with NF-κB activation, upregulates brain-derived neurotrophic factor (BDNF) expression, which affects gastric cancer cell sensitivity to anlotinib through the NTRK2/NFE2L2 pathway [62].

These intricate mechanisms highlight the critical role of CAFs in modulating tumor progression and therapeutic resistance, providing essential insights for developing novel therapies targeting the TME.

### Modulation of tumor immunity

Immune cells play a key role in the TME, including innate immune cells (e.g., macrophages, neutrophils, mast cells, MDSCs, dendritic cells (DCs), and natural killer [NK] cells) and adaptive immune cells (e.g., T and B lymphocytes). These cells play an important role in controlling and shaping tumor development [63]. CAFs interfere with T cell responses and are resistant to immunotherapy by secreting various immunosuppressive factors (e.g., IL-6, IL-10, C-X-C motif chemokine ligand 9 [CXCL9], and TGF-β) and recruiting suppressor immune cells (e.g., MDSCs and Tregs). In addition, the dense collagenous matrix produced by CAFs also affects immune cell infiltration[64, 65]. Therefore, targeting CAFs has become a new direction in anticancer immunotherapy [66, 67]. Two main strategies are currently used in cancer immunotherapy: (1) activating tumor antigens using cancer vaccines to stimulate immune cell responses to tumors and (2) enhancing endogenous antitumor activity by blocking immune checkpoint receptors, a strategy that involves targeting lymphocytes and their ligands [21].

#### Interaction between CAFs and immune cells

##### T cells

T cells, including CD4^+^and CD8^+^ subsets, are essential components of the adaptive immune system and play a pivotal role in defending against pathogens and tumors. However, CAFs profoundly influence T cell function and immune responses [69]. CAFs alter the physical and chemical properties of the stromal ECM through the secretion of collagen, hyaluronan (HA), and other factors, which impair T cell infiltration and tumor treatment efficacy. The deletion of type I collagen in myofibroblasts leads to increased CXCL5 production in tumor cells, promoting MDSC aggregation, reducing T and B cell populations, and accelerating cancer progression [66]. In prostate cancer, the expression of forkhead box F2 (FOXF2) in the stroma correlates with CXCL5 levels, and reduced CXCL5 enhances FOXF2 expression, leading to increased T cell infiltration [67]. Furthermore, the density of CAFs impacts the localization and abundance of CD8^+^T cells within tumors [70]. YAP1, a transcriptional coactivator secreted by myCAFs, inhibits CD8^+^ T cell infiltration, and its downregulation in HCC enhances CD8^+^T cell presence by reducing PD-L1 expression [68]. CAFs also secrete various chemokines, such as CXCL9, CXCL10, CXCL12, IL-6, and IL-8, to modulate T cell function. IL-6 promotes PD-L1 expression via the STAT3/AKT pathway, inducing T cell apoptosis and impairing immune responses [71, 72]. Similarly, IL-8 upregulates PD-L1 through NF-κB signaling, further inhibiting CD8^+^T cell activity [73].

Among CAF subtypes, mesothelial cell-derived CAFs (apCAFs) exhibit immune-regulatory functions and can induce CD4^+^T cell conversion to regulatory T cells (Tregs) under IL-10 and TGF-β influence [32]. Additionally, CAFs with enhanced metabolic activity (meCAFs), marked by PLA2G2A, regulate CD8^+^T cell function via the MAPK/ERK and NF-κB pathways, serving as prognostic markers for aggressive cancers [74]. TGF-β, a potent immune regulator secreted by CAFs, upregulates PD-L1 and Foxp3 expression, promoting Treg differentiation and inhibiting antigen-specific CD4^+^T cell expansion, thus impeding immune rejection and affecting chemo-immunological responses [75, 76]. In smmary, CAFs play a critical role in modulating T cell activity within the TME through various signaling pathways and secreted factors, influencing both tumor progression and immunotherapy outcomes.

##### NK cells

NK cells are also innate immune effector cells that can rapidly recognize and destroy abnormal and virus-infected cells in the body [77]. They are characterized by their ability to distinguish between normal and pathological cells and to recognize receptors by expressing multiple cell surface proteins [77, 78]. However, the immune function of NK cells is significantly affected by TGF-β, which, for example, leads to a decrease in the number of CD16-expressing NK cells in bladder cancer [79]. In human papillomavirus infections, NK receptor cell expression can be affected by influencing NK cell activation receptors such as natural cytotoxicity triggering receptors 3 (NCR3/NKp30) and 1 (NKp46) [80]. In addition, inhibiting TGF-β in NK cells was validated in a CRC model [81]. IL-6 and IL-8 secreted by CAFs decreased granzyme B (GZMB) in NK-92 cells [82]. Gene sequencing has identified a subset of senescent myofibroblasts (myCAFs) in breast cancer. These senescent myCAFs can affect NK cell function and foster tumor growth by secreting extracellular matrix ECM [83]. In addition, CAFs promote breast cancer bone metastasis by inhibiting NK cell activation and function through dickkopf-related protein 1 (DKK1) [84]. In gastric cancer, CAFs also lead to intracellular iron overload, triggering iron death in NK cells, and inhibiting this condition helped to enhance the efficacy of chimeric antigen receptor (CAR)-NK therapy [85]. CAR-NKcells can be designed based on molecules specifically expressed on CAFs to enhance the efficacy of cancer immunotherapy. For example, the specific expression of CD70 molecule (CD70) on CAFs can serve as a target for CAR-NK cells, with the CD70-CAR-NK cell interaction enhanced by IL-15 stimulation [86]. In addition, inhibiting IL-6 secreted by CAFs promotes enhanced CAR-NK cell function [87].

##### TAMs

As an important component of the TME, like other immune cells, TAMs interact with CAFs, which are critical for cancer progression and metastasis. Macrophages are usually classified into M1 and M2 types. The M1 type exhibits antitumor properties, eliminating tumor cells via phagocytosis and antibody-dependent cell-mediated cytotoxicity. In contrast, the M2 type has pro-tumorigenic effects, significantly impeding T-cell-mediated antitumor responses [88]. Single-cell sequencing has shown that various factors influence the gene expression profile of TAMs. For example, modulating SMAD3 reduced the potential for progenitor CAF production from macrophages [89]. The recruitment and polarization of TAMs are typically influenced by multiple factors secreted by CAFs, such as IL-8, IL-6, colony-stimulating factor 2 (CSF2/GM-CSF), and CXCL2 [90, 91]. CAFs promote tumor invasion and metastasis by promoting the release of FGF2 by secreting insulin-like growth factor-binding protein 7 (IGFBP7), which in turn affects the polarization of TAMs towards the M2 type and their infiltration into tumor tissue [92].

Lactate-activated CAFs can influence TAM recruitment by secreting IL-8 and contribute to TAM polarization toward the M2 type [93]. Additionally, CAFs draw monocytes through the CXCL12/C-X-C motif chemokine receptor 4 (CXCR4) signaling pathway and prompt their differentiation into M2-type macrophages, further promoting the formation of M2-type TAMs in oral squamous cell carcinoma [94]. One study examining the interaction between CAFs and TAMs found that G protein-coupled receptor 30 (GPR30) promoted macrophage recruitment and infiltration by upregulating CXCL12 and polarized more TAMs toward the M2 type with high IL-10 and low IL-12 expression [95]. Osteobridging protein (OPN), an intracellularly secreted chemokine-like phosphorylated glycoprotein, is involved in the interaction between CAFs and TAMs. Reducing OPN secretion from TAMs decreased OPN secretion by CAFs, and inhibiting OPN suppressed the proliferation, invasion, and migration of cancer cells induced by TAM-derived CAFs [96]. In addition, TAMs with activated Notch signaling expressed higher levels of immunosuppressive mediators, further enhancing their immunosuppressive function in the TME [97].

#### CAFs in energy metabolism-driven immune evasion of tumor cells

CAFs play a pivotal role in the modulation of TME and contribute to immune evasion mechanisms by altering cellular energy metabolism [98, 99]. Recent studies have highlighted that CAFs not only support tumor growth through secretion of growth factors and extracellular matrix remodeling but also significantly influence immune responses within the TME by manipulating metabolic pathways [100]. Tumor cells often adapt to a hypoxic, nutrient-deprived environment, reprogramming their energy metabolism to favor glycolysis and other anabolic processes, a phenomenon known as the Warburg effect [101]. CAFs interact with tumor cells through metabolic crosstalk, including the transfer of metabolites such as lactate, glutamine, and pyruvate, which not only sustain the energetic needs of tumor cells but also modulate the local immune landscape [102]. By secreting factors like IL-6, TGF-β, and CXCL12, CAFs create an immunosuppressive microenvironment, facilitating the recruitment and activation of immune cells such as Tregs and MDSCs, while inhibiting the function of CTLs and NK cells [101]. Moreover, CAFs contribute to metabolic reprogramming of immune cells, further enhancing immune escape by promoting the suppression of immune surveillance and effector functions [103, 104]. The metabolic cooperation between CAFs and tumor cells thus represents a critical mechanism through which tumors evade immune detection and destruction, providing novel insights for therapeutic strategies targeting the CAF-mediated metabolic reprogramming in cancer immunotherapy.

## Nanomaterials for targeting CAFs in cancer therapy

**Fig. 3:**
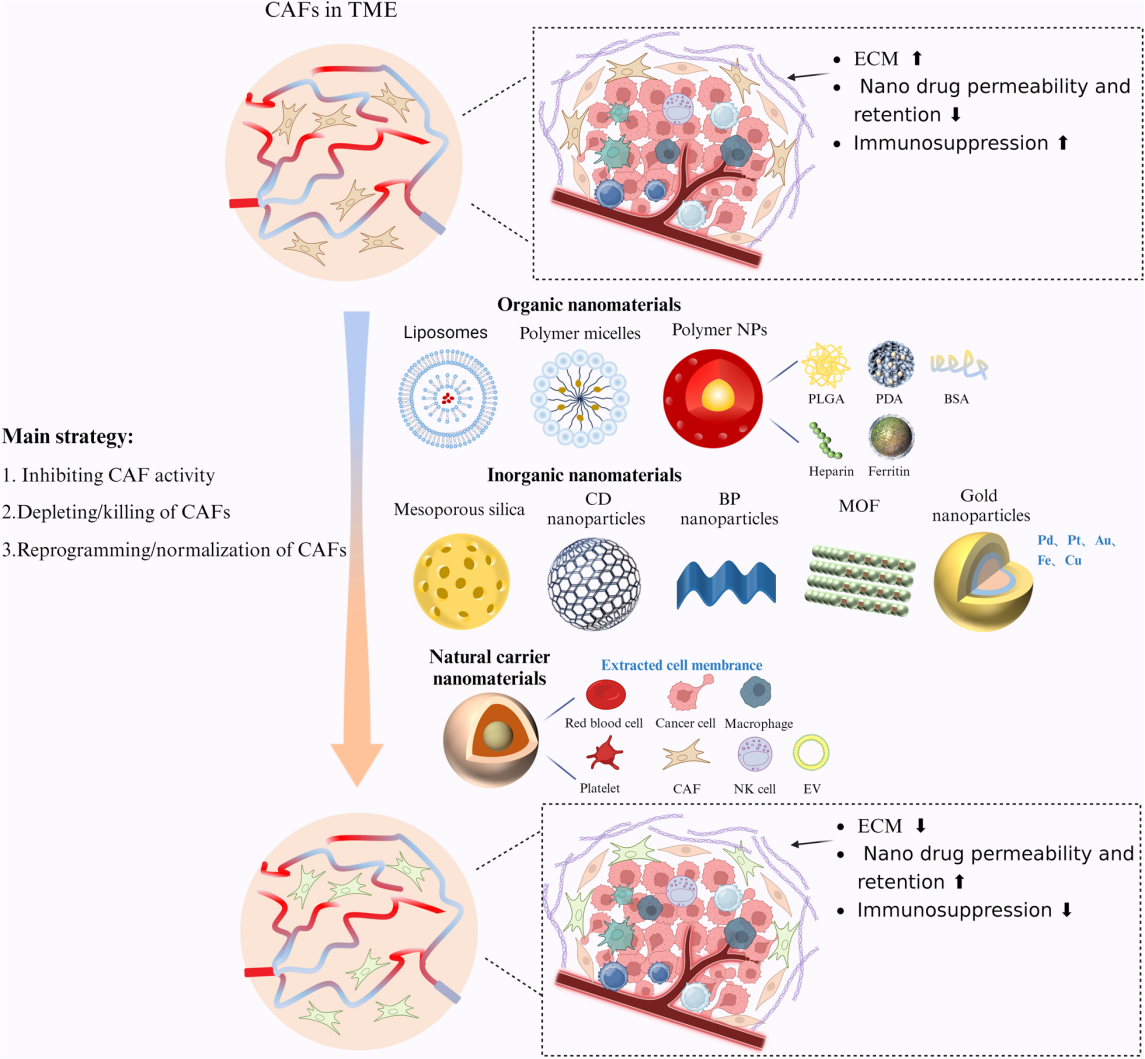
Functional nanocarriers for regulating CAFs and enhancing immunotherapy applications. By leveraging their unique physical and chemical properties, such as size, surface charge, and functionalization with targeting ligands, these nanocarriers can specifically interact with CAFs in the tumor microenvironment. This targeted approach not only helps in modulating CAF activity to overcome immunosuppressive barriers but also improves the delivery and efficacy of therapeutic agents

Increasing evidence underscores the pivotal role of CAFs in inducing immunosuppression within the TME, thus driving the development of CAF-targeted nanomaterials (Fig. 3). The TME is characterized by altered tissue architecture, a dense ECM, elevated interstitial fluid pressure, and hypoxia, all of which influence CAF behavior and the efficacy of nanomedicine delivery [105]. Moreover, tumor tissue stiffness, often resulting from ECM remodeling by CAFs, can impact signaling pathways and modulate the responsiveness of both CAFs and tumor cells to therapeutic interventions. Therefore, a comprehensive understanding of tumor physical properties, in combination with CAF-targeted strategies, is essential for optimizing the design of nanomedicines and enhancing therapeutic outcomes. Modulating tumor characteristics such as ECM stiffness or normalizing the vasculature may further improve the efficacy of CAF-targeted therapies [106].

In this section, we review various nanomaterials designed to modulate the TME for targeting CAFs and enhancing cancer treatment [107]. However, several challenges persist in utilizing nanomaterials for CAF modulation. These include difficulties in distinguishing between normal fibroblasts and CAFs, potential off-target effects, and issues with poor circulation stability, biocompatibility, and rapid clearance. To overcome these limitations, nanomaterials must be engineered with careful consideration of their physical properties (e.g., size, shape) and chemical properties (e.g., composition, surface chemistry) to enhance targeting specificity, stability, and biocompatibility. This section will explore the properties, advantages, and limitations of various nanomaterials that aim to improve cancer immunotherapy by regulating CAFs. Table 1 summarizes key nanomaterials that enhance immunotherapy outcomes through CAF targeting.

**Table 1:** Applications of nanomaterials in targeting CAFs to enhance immunotherapy

Nanomaterial	Component	Reagent	CAFs-targeting ligand	Stimulus–response	Cancer model	Refs
Polymer micelles	PEG-PBLG	Epirubicin/ Tranilast/DOX	/	/	Breast cancer	[108]
	mPEG-hyd-PLGA/Cy5-PEG-hdy-PLGA	PTX/AMD3100/BMS-1	/	pH	TNBC	[109]
	COOH-PEG-CPAA	DOX/Talabostat mesylate	/	pH/ROS	Breast cancer	[110]
	mPEG 2 K	LY2157299	FAP-α	ROS	Pancreatic cancer	[111]
Liposomes	PAMAM dendritic macromolecule/DOPE/DSPE-PEG	DOX/R848/Lorsatan	/	pH	Breast cancer	[112]
	Phosphatidylcholine/Cholesterol	Silybin/DOX	/	/	Breast cancer	[25]
	DSPE-PEG2K-ATF/mPEG-DSPE	Cisplatin	uPAR	pH	Pancreatic cancer	[113]
	DSPE-PEG2K/Cholesterol/Lecithin	Salvianolic acid B	/	/	Breast cancer	[114]
Polymeric NP	DSPE-PEG2K	Larotaxel/ Cetyl alcohol	/	GSH	Breast cancer	[115]
	DPPC/DSPE-PEG2K OMe/MSPC	BMS-202	/	FAP-α/NIR	Pancreatic cancer	[116]
	PEI25K-PPhe(PF180)	Quercetin	/	pH	Breast cancer	[117]
	PLGA/PVA	Losartan/Metformin	/	/	Melanoma	[118]
	PEG-PLGA	Baicalein	/	/	TNBC	[119]
	DnPEA-mPEG-PAMAM dendritic macromolecule	Gemcitabine	/	pH	Pancreatic cancer	[120]
Si-based NP	Silica	siRNA	/	/	Liver cancer/Pancreatic cancer/Colorectal cancer	[121]
C-based NP	NH_2_-PEG5K-NH_2_/PEG-CDs	DOX/ Losartan	Asp-Ala-Thr-Gly-Pro-Ala Peptides/AEAA	FAP-α	Breast cancer	[122]
Phosphorus-based NP	N-Methyl-2-Pyrrolidone/BP Crystal	SCH442416/The A2A receptor	/	NIR	Melanoma	[123]
Metal-NPs	Fe_3_O_4_/AuNP/ PLGA	DOX/SPIO	/	/	Breast cancer	[124]
	Cu(II) based MOF	Blebbistatin/FAP-α targeted peptide	/	FAP-α	Breast cancer	[125]
	HF based nMOF	Anti PD-L1 antibody	/	/	Pancreatic cancer	[126]
	Fe_3_O_4_ based MOF	Oxymatrine/Astragaloside IV/Anti PD-L1 antibody	/	/	Liver cancer	[127]
	FeCo-ZIF/PEG2K-NH_2_	Panobinostat/Telmisartan	/	/	Breast cancer	[128]
Natural carrier	Human cancer cell membrane/PLGA	/	/	/	Melanoma	[129]
	Cancer cell membrane/CAFs membrane/Cholesterol	Paclitaxel/PFK15	/	/	Breast cancer	[130]
	4T1/Cancer cell membrane/CAFs membrane	Pirfenidone/DOX	/	/	Breast cancer	[131]
	Red blood cell membrane/PLGA	Silybin	/	/	Breast cancer	[132]
	Platelet membrane/Fe_4_O_3_	Oxymatrine/Astragaloside IV	/	/	Liver cancer	[127]
	Exosomes/DOPE/DSPE-PEG2K-AEAA	JQ1/C18/IL-12 plasmid ceramide/Gemcitabine	/	/	Pancreatic cancer	[133]

### Organic nanomaterials

Organic nanomaterials offer significant advantages in cancer immunotherapy due to their unique physicochemical properties and tunable compositions, which make them powerful tools for enhancing the efficacy of immunotherapeutic approaches. Common examples of organic nanomaterials include liposomes composed of phospholipid bilayers, NPs made from synthetic or natural polymers, microsphere emulsions formed by crosslinked polymers, and polymeric microspheres [19, 134]. These materials not only enable targeted drug delivery within the TME, but also modulate immune responses, thereby improving the therapeutic outcomes of immunotherapy.

The physicochemical characteristics of organic nanomaterials, such as adjustable particle size, surface properties, and structure, provide precise control over drug delivery and release. By encapsulating anticancer drugs or immune modulators within these nanocarriers, they can effectively overcome biological barriers and deliver drugs directly to the tumor site. This approach can inhibit the activity of CAFs or reduce the secretion of immune-suppressive factors by CAFs, further enhancing therapeutic efficacy. A thorough understanding of these properties is crucial for designing more precise and effective therapeutic strategies.

#### Polymer micelles

Micelles are nanoscale colloidal particles formed through the self-assembly of amphiphilic polymers. In solution, polymer concentration plays a crucial role in micelle formation[135, 136]. Below the critical micelle concentration (CMC), polymers exist as individual molecules, whereas above the CMC, they aggregate into micelles, which have a hydrophobic core and a hydrophilic shell. Common hydrophilic components of polymeric micelles include polyethylene glycol (PEG) and polyvinyl alcohol (PVA), while the hydrophobic components are often made from materials such as polylactic acid (PLA), polycaprolactone (PCL), polyether, or polylactic-glycolic acid copolymer (PLGA) [137, 138]. These materials have found widespread application in nanoplatforms designed to modulate CAFs and enhance immunotherapy. For example, Myrofora et al. used PEG-b-poly(benzyl-L-glutamate) (PEG-PBLG) block copolymers to prepare polymeric micelles loaded with Tranilast [108]. These micelles had an average size of approximately 95 nm and demonstrated high stability, retaining their original size even after prolonged storage, with no precipitation observed in solution. Similarly, in a study by Feng, PEG-PLA was used to target CAFs [137].

To improve the affinity of polymeric micelles for CAFs and enhance the effectiveness of targeted tumor therapies, several strategies have been employed. One such strategy involves incorporating CREKA peptides, which specifically bind to fibronectin overexpressed on CAFs [137], or peptides targeting FAP-α, such as Gly-Pro-Ala-Cys and Ac-RQRQGPA-OH [111, 138]. In addition, Cheng et al. demonstrated that polymeric micelles could non-covalently bind to antibody fragments for CAF targeting [139]. Furthermore, polymeric micelles can also complex with siRNA via electrostatic interactions, thereby facilitating CAF uptake. For example, in the work of Paul et al. a triblock polymeric micelle was designed, with poly(sarcosine) (pSar) as the first block to provide stealth characteristics and avoid rapid blood clearance and immune recognition, poly(γ-benzyl-L-glutamate) (pGlu(OBn)) as the second block to stabilize the micelle structure, and poly(L-lysine) (pLys) as the third block, which complexes with siRNA through electrostatic interactions [140].

With the continuous progress in nanotechnology, an increasing number of multifunctional polymeric micelles have been developed to respond to changes in the TME, such as variations in temperature, pH, redox state, and enzyme activity. This enables precise drug release at the tumor site [141]. Hyaluronic acid (HA), a natural high-molecular-weight polysaccharide known for its excellent biocompatibility and biodegradability, is also a promising material for synthetic polymeric micelles. For instance, in the research of Liang et al., HA was deacetylated to form a zwitterionic polysaccharide, which was then hydrophobically modified by grafting dodecylamine to create a pH-sensitive zwitterionic polymer [138]. In this case, the hydrophobic modification with dodecylamine enhanced the hydrophobicity of the micelles, thereby promoting the formation of a stable micelle structure.

#### Liposomes

Liposomes are vesicular structures composed of one or more phospholipid bilayers, characterized by a hydrophilic interior and a hydrophobic exterior [142, 143]. These properties enable liposomes to effectively encapsulate both hydrophilic and hydrophobic drugs [144, 145]. Common materials used in liposome construction include Phosphatidylcholine, Cholesterol, and Egg yolk lecithin. These components play a critical role in drug delivery systems by contributing to the formation of stable NPs and enhancing drug bioavailability and targeting capabilities [25, 144]. The therapeutic efficacy of liposomes is closely related to their internal stability, which is influenced by several factors such as particle size, charge, the number of membrane layers, the presence of targeting ligands, and the specific materials used in their construction [145, 146].

Liposome preparation methods are relatively straightforward, with common techniques including the film hydration and ethanol injection methods [114, 144]. For example, Wang et al. developed liposomes composed of DOPC, Cholesterol, and DSPE-PEG, loading drugs using the film dispersion method. These liposomes, with an average size of approximately 100 nm, demonstrated effective biodistribution in the bloodstream and within cells, and exhibited good physical stability with minimal changes in particle size after storage at 4 °C for 20 days [147].

However, liposomes are often rapidly recognized and cleared by the mononuclear phagocyte system (MPS), which can limit their effectiveness [148]. To address this challenge, modifications are often made to evade MPS recognition. For example, Chen et al. introduced hydrophilic polymer modifications, such as Polyethylene Glycol (PEG), to prolong the circulation time of drugs [114]. In addition, researchers have further optimized liposome compositions to improve targeting. For instance, ATF peptides specifically bind to Upar [149], CFH peptides show high affinity for Tenascin-C [150], and peptides with strong binding affinity for fibronectin and type I collagen, such as FnBPA5, can also be incorporated into liposomes [151].

To better respond to the dynamic TME, researchers have incorporated stimuli-responsive materials into liposomes. These materials allow for the specific release of drugs at the tumor site, reducing exposure to normal tissues and potentially minimizing side effects. For example, Yu et al. developed pH-sensitive liposomes by combining DSPE with PEG2K [113]. These liposomes, when delivering chemotherapeutic drugs to the tumor site, suppress CAF activation, overcome tumor stromal barriers, and promote immune cell infiltration. Additionally, drug release can be triggered by temperature changes. Tan et al. utilized dipalmitoylphosphatidylcholine (DPPC) as part of thermosensitive liposomes, which encapsulated near-infrared photosensitizer IR780 iodide and BMS202 [19]. Upon laser irradiation, the liposomes undergo a gel-to-liquid crystal phase transition, accelerating the disintegration of the lipid bilayer structure and disrupting the ECM and CAFs, thus promoting the infiltration of tumor-infiltrating lymphocytes (TILs). This thermosensitive nanoliposome achieved a drug loading (DL%) of 7.0% and an encapsulation efficiency (EE%) of 77.6%.

Through optimization of liposome preparation processes, surface modifications, and the integration of multifunctional capabilities, liposomes can significantly improve drug stability, targeting precision, and the effectiveness of immunotherapies. These advancements hold great promise in overcoming the challenges posed by immunosuppressive cells, such as CAFs, in the tumor microenvironment.

#### Polymer NPs

Polymeric NPs have emerged as a powerful tool in cancer immunotherapy, offering significant advantages for targeted drug delivery [21, 134]. These NPs are generally classified into two main types: nanocapsules and nanospheres [152, 153]. Nanocapsules encapsulate drugs within an oily core, surrounded by a polymeric shell that controls drug release. This design reduces non-specific drug distribution and enhances targeted therapy. In contrast, nanospheres achieve drug stability by adsorbing or entrapping drugs within a polymer network. Both types are typically composed of synthetic or natural polymers, which provide excellent biocompatibility, biodegradability, accessibility, and cost-effectiveness [26, 154, 155].

Common synthetic polymer materials include novel nucleotides PEI25k-PPhe (PF180), poly(lactic-co-glycolic acid) (PLGA), poly(ethylene glycol)-block-poly(D,L-lactic acid) (PEG-PLA), and 1,2-distearoyl-sn-glycero-3-phosphoethanolamine-N-poly(ethylene) (DSPE-PEG) [121, 122, 155]. These amphiphilic polymers are designed to improve the TME by modulating CAFs and enhancing the efficacy of immunotherapy. For instance, PLGA, which is known for its favorable physicochemical properties, preferentially accumulates in tumor tissues while avoiding unwanted immune activation due to its immune-inert nature. PLGA NPs, with a zeta potential between − 35 mV and − 50 mV, have been shown to induce apoptosis in CAFs and alter the phenotype of TAMs [134]. PEGylated PLGA NPs provide stealth functionality, thereby extending circulation time and reducing clearance by the RES [119]. Huang et al. developed PLGA-based NPs to regulate CAF-secreted cytokines, such as CAA and CCL2, thus inhibiting tumor growth and improving the TME [156]. Similarly, Jiang et al. demonstrated that NPs made from the AEAA-PEG-PCL polymer carrier significantly enhanced drug solubility and targeting after systemic administration [157]. These NPs are spherical and relatively uniform in size.

Cationic polymers, such as PolyMet, have gained attention for their high gene transfection efficiency and low toxicity. For example, Zhang et al. showed that PolyMet can effectively deliver plasmids encoding Relaxin, protect DNA from dissociation, and improve TGF-β-induced CAF activation, thereby altering the immunosuppressive microenvironment [158]. Polydopamine, a biocompatible material, has been used as a coating for NPs, enhancing the matrix barrier mediated by CAFs through photothermal effects [159, 160].

Hydroxyethyl starch-folate conjugates (HES-FA) are amphiphilic surfactants that achieve high drug loading efficiency and tumor selectivity. By utilizing isothiocyanate-bridged doxorubicin dimer prodrugs, HES-FA can reduce CAF activity [161].Dendritic polymers also play an important role in drug delivery. For instance, Zhang et al. conjugated dasatinib (DAS) to dendritic poly(oligo(ethylene glycol) methyl ether methacrylate) (POEGMA) via a tetrapeptide linker to create dendritic polymer NPs designed for modulating CAFs. Additionally, they linked epirubicin (Epi) to dendritic POEGMA using acid-responsive hydrazone bonds to facilitate ICD induction [162].

Natural polymers, such as bovine serum albumin (BSA), heparin, gelatin, chitosan, and cellulose, are increasingly used due to their safety and stability [21, 117, 159, 160]. In a study by Hua et al., BSA encapsulated quercetin via hydrophobic interactions and hydrogen bonding to inhibit CAF activity, reshape the TME, and improve nanoparticle penetration and immune cell infiltration [117].Acetylated methylcellulose exhibits excellent biocompatibility and drug delivery properties, aiding in nanoparticle accumulation in the tumor stroma by interacting with SMA-positive fibroblasts and F4/80-positive macrophages [163]. Heparin, with its favorable compatibility, degradability, and water solubility, can target heparanase—a protein highly expressed in tumors—offering a novel strategy to alleviate CAF-mediated immunosuppression [13].

Moreover, polymeric NPs can be engineered to respond to TME-specific characteristics, thereby improving CAF function and enhancing the efficacy of immunotherapy. Examples include glutathione (GSH)-triggered, reductive-sensitive nanoparticles [118, 120, 157]. heparinase- and pH-responsive nanoparticles [115], heparinase- and pH-responsive nanoparticles [13], and FAP-α-influenced self-assembling NPs [17].

### Inorganic nanomaterials

Inorganic nanomaterials exhibit unique physicochemical properties that make them highly promising for biomedical applications, particularly in cancer therapy, diagnostics, and immunotherapy. These materials, including black phosphorus (BP), silicon-based, metal-based, metal-oxide-based, metal–organic frameworks (MOFs), and self-assembled nanomaterials, offer significant advantages in drug delivery, imaging, and therapeutic targeting. For example, the layered structure of BP enhances its performance in PTT and photosensitive diagnostics. Silicon-based nanomaterials, known for their stability and tunability, improve drug delivery by enhancing biocompatibility and targeting precision. Metal-based nanomaterials, such as gold and silver, are particularly effective in optical imaging and PTT, while metal oxide nanomaterials, like manganese and iron oxide, excel in MRI imaging and thermal therapy [164]. These properties not only improve the efficacy of conventional treatments but also help overcome tumor immune evasion, offering substantial potential for advancing cancer therapy and immunotherapy.

#### Silicon-based nanomaterials

Silicon, one of the most abundant elements on Earth, plays a critical role in the diagnosis and treatment of cancer[165, 166]. Solid silicon dioxide and mesoporous silicon dioxide (DMSN) nanostructures, known for their excellent chemical stability, thermal stability, and mechanical robustness, have found widespread application in cancer therapeutics [167, 168, 169]. The superior biocompatibility and blood compatibility of DMSN make it an ideal candidate for constructing drug delivery systems, thereby offering multiple avenues for cancer treatment. Additionally, its large surface area and high porosity create favorable conditions for drug loading and controlled release [170]. For example, in He’s research, DMSN was combined with trypsin-like protease imprinted polymers to neutralize trypsin-like protease (TPS), resulting in changes to the phenotype of CAFs and enhancing the efficacy of tumor immunotherapy [155].

In terms of synthesis, hybrid organic–inorganic approaches can be utilized to prepare hollow mesoporous organosilica NPs. These nanostructures not only enable efficient drug loading, but with surface modifications such as diselenide coupling, they also facilitate the degradation of the silicon-based material under the influence of reactive oxygen species (ROS), thus promoting the release of encapsulated drugs. This process not only supports the effective release of therapeutic agents but also triggers a potent immunogenic response. Moreover, binding to PD-L1 on the surface of tumor cells further amplifies the systemic immune response [171]. Furthermore, DMSN has been shown to load negatively charged siRNA, effectively shielding it from degradation and thereby influencing the behavior of CAFs [121].

#### Carbon-based nanomaterials

Carbon-based nanomaterials, including graphene, carbon quantum dots, and carbon nanotubes, have shown great potential in cancer therapy and diagnostics. Their unique properties make them ideal for drug delivery systems in cancer immunotherapy [172]. Surface modifications, both covalent and non-covalent, can enhance drug targeting and improve anticancer efficacy [168].

Carbon-based nanomaterials exhibit excellent optical properties in the near-infrared (NIR) region, making them ideal for photothermal therapy, which can trigger strong antitumor immune responses. Their biocompatibility and water dispersibility also make them excellent fluorescence imaging probes for early cancer detection and monitoring. For instance, carbon dot(CD) nanoclusters serve as imaging agents, photothermal therapy tools, and carriers for chemotherapeutic drugs and immunological inducers, playing a key role in cancer treatment and immunotherapy [173, 174, 175].

The high surface area and porous structure of carbon dots enhance their ability to adsorb chemotherapeutic drugs and immunological adjuvants. In a study by Hou et al. DOX molecules were efficiently loaded onto CDs through π-π stacking interactions with the large conjugated π system of CD, facilitating the drug’s binding to the nanomaterial [122]. Furthermore, the study involved surface modification of CDs with AEAA-PEG-NH₂ and NH₂-PEG-NH₂, which endowed the CDs with the ability to specifically target CAFs and significantly improved their stability. Additionally, graphene-based fluorescence NPs, synthesized via hydrothermal reactions, achieved labeling efficiencies of up to 60% for CAFs [176].

#### BP-based nanomaterials

BP, as a member of the two-dimensional material family, is a unique metal-free layered semiconductor. Its bandgap is tunable, capable of flexibly shifting from 0.3 eV in bulk material to 2.0 eV in monolayer materials, and it also possesses ultraviolet and near-infrared light absorption characteristics. These exceptional optical properties give it immense potential in the field of cancer therapy, especially in optical treatment, drug delivery, and diagnostics [174, 177].

Compared to other two-dimensional materials such as graphene and MoS, black phosphorus nanosheets exhibit higher drug loading capacity and significant pH/photon response capability [173]. Their good biocompatibility and photostability further establish their position as an ideal choice for a multifunctional nanoplatform. Surface modification of BP through physical or chemical means can achieve multiple goals, such as active targeting, extended retention time in the body, enhanced photosensitivity, and improved photothermal conversion ability [178, 179].

In practical cancer treatment scenarios, black phosphorus has shown excellent results in both PTT and PDT. When exposed to specific laser irradiation, it not only effectively inhibits tumor growth but also strongly activates antitumor immune responses [180]. More importantly, bioactive black phosphorus can suppress the activation of CAFs, subtly promoting the interaction between tumor and stroma, thereby further enhancing the overall therapeutic effect [180]. Additionally, black phosphorus can act as a bridge to synergize the combined impact of photothermal therapy and adenosine blockade, significantly improving the physical barrier of tumor stroma and immune-suppressive factors and thereby considerably enhancing the activity of cytotoxic T cells [123]. However, BP is highly susceptible to oxidation, and it is commonly modified, for example, by loading with PEG [178].

#### Metal-based nanomaterials

Metal-based nanomaterials have attracted much attention due to their unique biological effects [181]. The commonly used elements are calcium (Ca^2+^), manganese (Mn^2+^), iron (Fe^2+/3+^), and potassium (K), which can be modified to synthesize a wide range of common metal-based nanomaterials, such as CaCO_3_ NPs, manganese oxide (MnO) NPs (e.g., MnO, MnO_2_), FeO NPs (e.g., Fe_3_O_4_), and NaCl NPs [179, 182]. These materials modulate immune responses in tumor immunotherapy by triggering and influencing key immune processes. For instance, FeO NPs contain both Fe^2^⁺ and Fe^3^⁺ ions, with Fe^2^⁺ promoting the innate immune response against cancer cells by catalyzing the Fenton reaction to generate ROS, which can induce classical cell death and ICD. In contrast, Fe^3^⁺ enhances the effects of ICD by preventing ROS clearance, a process that involves depleting excess GSH. Additionally, Fe ions can stimulate macrophages to adopt the immunostimulatory M1 phenotype [183].

Moreover, metal-based nanomaterials can also exhibit photothermal effects, which can further enhance their therapeutic potential [183, 184].For example, He et al. developed Fe/Co bimetallic nanocarriers that not only trigger the Fenton reaction to induce ferroptosis but also possess photothermal properties. This dual-functionality enables targeted therapy of both tumor cells and CAFs [128].

In another study, Zheng et al. utilized gold nanoparticles (Au NPs) modified with polyallylamine hydrochloride (PAH) to alter the surface charge of the Au NPs, converting it from negative to positive. This modification enabled the Au NPs to bind to the surface of Fe₃O₄ NPs. The combination of photothermal therapy and imaging significantly impacted CAFs, enhancing ICD and activating immune responses [124]. In addition to iron oxide, other metal elements such as palladium (Pd), platinum (Pt), and gold (Au) also play crucial roles in tumor immunotherapy. For example, Hou et al. designed PdPtAu NPs with a metal-polyphenol network on their surface [185]. These NPs, with a spherical shape and an average diameter of 75 nm, exhibited a well-defined mesoporous structure.

MOFs, as metal-based nanomaterials with three-dimensional porous structures, have garnered significant interest in recent years due to their promising applications in biomedicine. MOFs are typically composed of inorganic metal nodes or clusters, offering advantages such as excellent biocompatibility, chemical stability, tunable porosity, and versatile surface modification capabilities, making them well-suited for biomedical purposes [184, 185, 186]. These materials have the potential to elicit robust immune responses by encapsulating antigens and immune adjuvants. For example, MOF surfaces can selectively bind to cytosine-phosphate-guanosine (CpG) oligonucleotides, thereby triggering immune activation [187, 188]. Furthermore, MOFs can be used to deliver PD-L1 inhibitors, which counteract immunosuppressive signals and enhance the efficacy of immunotherapy [189].

In research by Guo et al. a MOF based on Fe₄O₃ was coated with a platelet membrane [127]. MOFs can also be combined with metals like copper (Cu) and iron (Fe). For example, Meng et al. developed Cu(II)-MOFs that encapsulated Blebbistatin and a FAP-responsive peptide (Tp), enabling targeted delivery to CAFs and photothermal triggering [125]. Huang et al. synthesized a MOF that coordinated Fe^3^⁺ with PLGA nanocores, forming a stable, negatively charged MOF structure on the surface. This structure reduces drug molecule collisions, delays aggregation and sedimentation of drugs in solution, and enhances colloidal stability [187]. This MOF encapsulated Erastin, forming a spherical core–shell structure with a particle size of approximately 170 nm and a shell thickness of around 30 nm. It also promoted M1 macrophage polarization while reducing TGF-β secretion.

Overall, these metal-based nanomaterials are emerging as powerful tools in cancer immunotherapy due to their ability to modulate immune responses, induce cell death, and enhance the efficacy of immunotherapeutic strategies. Their multifunctionality, along with the potential for surface modifications, positions them as promising candidates for targeted cancer therapies and immune modulation.

#### Natural carrier nanomaterials

Cell membranes, as natural carrier nanomaterials, consist of lipid bilayers and are adorned with proteins, glycoproteins, and lipoproteins on their surface[190]. These molecules play crucial roles in immune evasion, homing targeting, and regulation of inflammatory responses [191, 192]. Researchers have exploited these properties by camouflaging nanoparticles (NPs) with cell membranes, thus mimicking the antigenic diversity of the original cells and endowing the NPs with functionalities akin to those of the original cell membrane [193]. Cell membranes from various sources—such as erythrocytes, tumor cells, macrophages, and NK cells—have been widely utilized to coat NPs, enhancing their ability to modulate CAFs.

To further augment the functionality of these membranes, coupling methods involving amines, carboxyl groups, and thiols are often used to decorate the cell membranes with functional ligands [191, 194]. For example, Li et al. developed hyaluronic acid (HA)-polyethylene glycol (PEG)-lipid conjugates for surface engineering of ex vivo NK cells, thereby enhancing the efficacy and duration of NK cell membrane coating [192]. This approach does not induce off-target effects on human fibroblasts, boosting the effectiveness of NK cells in cancer-targeted immunotherapy. In the field of cancer nanotherapy, biomimetic cell membrane technology has been increasingly used to improve immune evasion in immunotherapy by mimicking immune cells in the inflamed tumor microenvironment [193].

Macrophage membranes, for instance, can recruit additional immune cells to inflammatory sites by releasing chemokines and cytokines, thereby activating immune responses [195]. This makes macrophage membranes an excellent choice for drug delivery, especially when targeting tumor cells, as they significantly improve drug targeting, biocompatibility, and reduce unwanted immune responses [194].Du et al. utilized macrophage membranes encapsulating α-mangostin and O_2_ to form biomimetic nanoprobes, enhancing the ECM created by CAFs, Improving drug penetration, and facilitating immune cell infiltration [196].

In some studies, homing targeting strategies are employed, such as coating NPs with activated fibroblast membranes to minimize immunogenic responses and effectively target CAFs, thereby improving the tumor microenvironment [197]. For example, Jia et al. constructed liposomes coated with CAF and tumor cell membranes, leveraging the inherent homing properties of these cell membranes to specifically target CAFs and tumor cells [131]. Similarly, Zang et al. used CAF and tumor cell membrane-coated solid lipid nanoparticles [131].Genetic engineering techniques can also be combined with biomimetic nanocarriers to introduce molecules that specifically bind to the FAP receptor on CAFs, enabling precise targeting and elimination of CAFs, while triggering immune responses against tumors [198].

Erythrocyte and platelet membranes, the two major types of cell membranes in blood, have also been applied to regulate CAFs. Erythrocytes are particularly advantageous for extraction and purification because they lack nuclei and other organelles. Furthermore, they are highly effective in evading phagocytosis by monocytes and macrophages, assisting NPs in targeting CAFs. Guo et al. coated MOFs with platelet membranes, which specifically recognize and adhere to collagen secreted by CAFs in the tumor microenvironment [132].

Additionally, extracellular vesicles (EVs) derived from cell membranes have gained significant attention in tumor immunotherapy. These vesicles serve as ideal drug delivery vehicles due to their role in extracellular communication between tumor cells and CAFs. For example, Yuan et al. demonstrated that extracellular vesicles help drugs cross the tumor stromal barrier and enhance the immune response against tumors [133]. Hu et al. explored exosome-like nanovesicles derived from FAP gene-engineered tumor cells [199].

### Comparison of different nanoparticles

Each type of nanomaterial possesses unique advantages and limitations within drug delivery systems. When selecting nanomaterials for drug delivery, it is essential to consider their comprehensive biocompatibility, stability, drug loading capacity, targeting ability, and biosafety. Through further research and optimization, these nanomaterials hold the potential to play an increasingly significant role in future drug delivery systems. Table 2 will provide a comparison of the nanomaterials listed above.

**Table 2:** Comparison of Different Nanomaterials

Nanomaterials type	Composition and structure features	Advantages	Limitations	Ref
Polymer Micelles	Amphiphilic polymers self-assemble, including hydrophilic and hydrophobic parts	Improvement of drug solubility and stability; high-efficiency gene delivery capability; enhanced cellular uptake and endosomal escape (at the nanoscale); good biocompatibility and safety; multifunctionality	The synthesis and preparation processes are complex; the loading capacity for specific drugs or molecules is limited; the in vivo behavior exhibits complexity and uncertainty; and there are significant challenges in clinical translation	[108, 138, 140, 200]
Liposomes	Formed by single or multiple lipid-based bilayers	Capable of encapsulating both water-soluble and lipophilic drugs; efficient drug delivery and synergistic effects; excellent biosafety; targeted delivery potential	Individual differences in in vivo distribution; conventional liposomes are prone to recognition and uptake by the MPS	[114, 147, 201, 202]
Nanoparticle Polymers	It can be composed of natural materials or synthetic polymers	Efficient drug delivery and gene transfer; good design flexibility enables control over drug release kinetics; excellent biocompatibility	Potential long-term toxicity; the preparation process is complex and requires optimization	[158, 203, 204]
Carbon-based Nanoparticles	Including carbon nanotubes, graphene, fullerene, etc	High drug and metal loading capacity; excellent biocompatibility; low cytotoxicity and ease of surface functionalization; high photothermal conversion efficiency	The small particle size facilitates rapid clearance from the circulatory system (e.g., CDs < 20 nm)	[122, 205]
Silicon-based nanomaterials	It can be composed of solid silica and mesoporous silica (DMSN)	Good biocompatibility and blood compatibility; large specific surface area and high porosity, conducive to drug loading and release	–	[155, 166, 165, 170]
BP-based nanomaterials	Metal-free layered semiconductor composition	Exhibits excellent NIR-II photothermal conversion efficiency; can be used as a PTT photosensitizer and self-delivering drug nanocarrier. Demonstrates biocompatibility and biodegradability	Prone to oxidation	[123, 178]
Metal-based Nanoparticles	Fe_3_O_4\_Au, etc	Excellent photothermal performance; stability and drug loading capacity; multimodal combination therapy; trigger of Fenton reaction	In vivo stability and potential bio-safety issues, as well as high-dose injection, need caution	[124, 128, 185]
	MOF	Good photothermal effect; large surface area; tunable structure		[125, 127, 206]
Biomimetic Nanoparticles	Cell membranes (e.g., erythrocyte membranes, macrophage membranes, etc.); cell microparticles	Good biocompatibility can prevent immune evasion and improve drug targeting	–	[194, 196, 198, 207]

## Mechanisms of targeting distinct CAFs

**Fig. 4:**
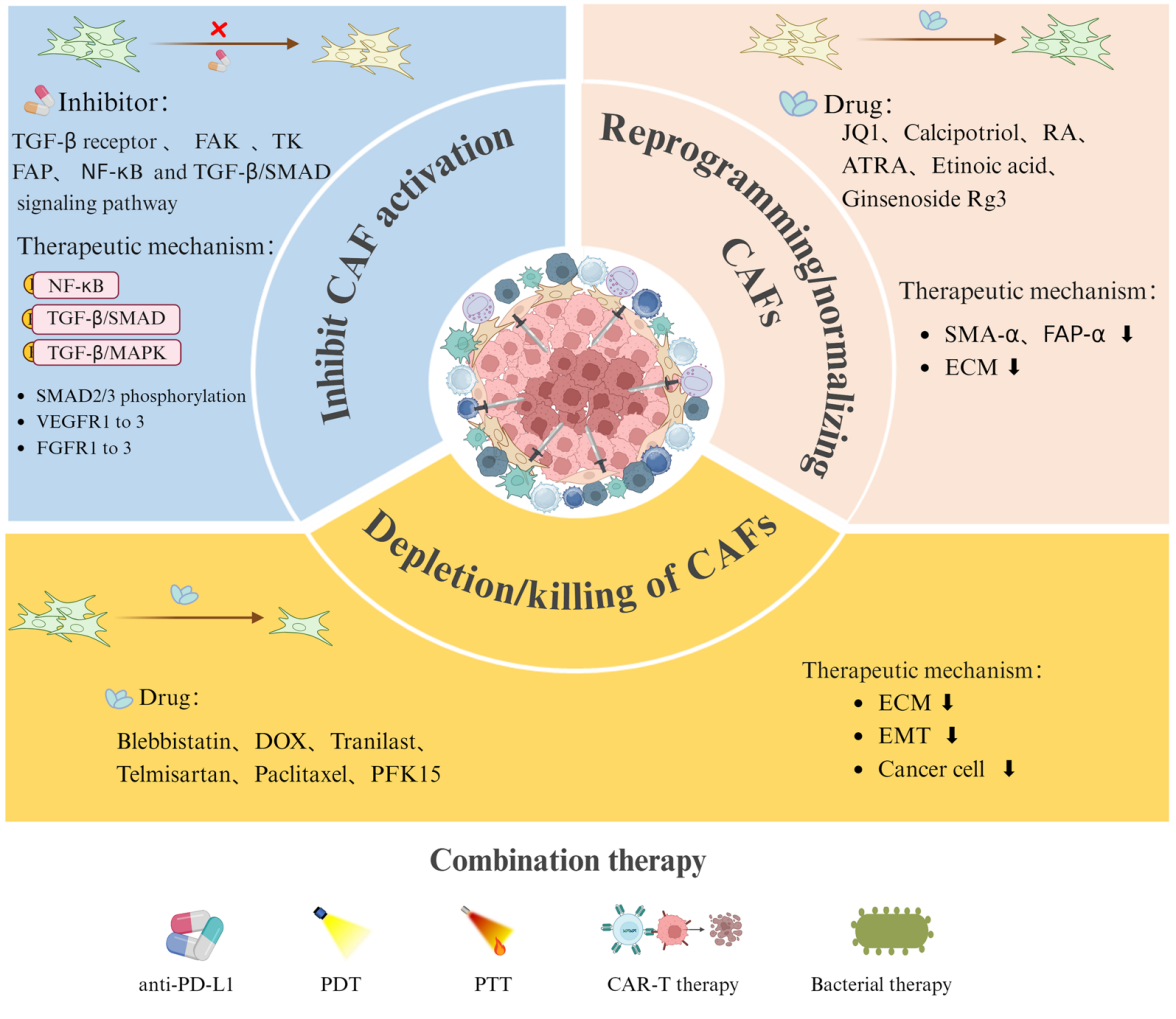
The current main regulatory approaches of CAFs and combination therapies

A comprehensive understanding of the regulatory mechanisms that govern CAF functions is crucial for the development of more effective therapeutic strategies (Fig. 4). Furthermore, the integration of nanodrugs with complementary modalities, such as photothermal therapy (PTT) and photodynamic therapy (PDT), offers significant promise for enhancing therapeutic outcomes. In this section, we examine the current mechanisms by which targeting CAFs can be utilized to advance cancer treatment, providing valuable insights into the development of more efficient therapeutic approaches. Table 3 outlines the mechanisms through which targeting CAFs can optimize tumor immunotherapy outcomes.

**Table 3:** Mechanism of action of nanomedicines targeting CAFs

Strategy	Nanomaterial	CAFs-targeting reagent	Therapeutic mechanism	Cancer type	Refs
Inhibition CAF activation	MOF	Oxymatrine	Inhibition of CAF activation by reversing EMT and increasing the level of TILs	Liver cancer	[127]
	Liposome	Pirfenidone/DOX	Inhibition of CAFs by inhibiting pro-fibrotic cytokines and collagen synthesis	Breast cancer	[131]
	Polymer NP	Curcumin	Baicalein reverses cellular fibrosis and reduces the expression of immunosuppressive factors by inhibiting the activation of the TGF-β/SMAD pathway and the TGF-β / MAPK pathways	Breast cancer	[119]
	Polymer NP	Galunisertib	Galunisertib inhibited SMAD2/3 signaling by inhibiting phosphorylation and inducing immunosuppression	Colorectal	[9]
	Polymeric micelles	Talabostat mesylate	Induction of ICD and promotion of TNF-α secretion and T-lymphocyte infiltration by inhibiting TGF-β secretion and downregulating α-SMA expression	Breast cancer	[110]
	Nano Pue	Puerarin	Effective inhibition of SMAD2/3 phosphorylation and TGF-β/SMAD pro-fibrotic signaling by downregulating ROS in activated fibroblasts, thereby affecting immunosuppressive factors	Breast cancer	[208]
	Polymeric micelles	Valsartan/DOX	Enhanced drug penetration and immune cell infiltration by decreasing type I collagen and α-SMA expression	Breast cancer	[138]
	Nanogel	Oleanolic acid	Modulation of the ECM by attenuating fibrosis by inhibiting TGF-β/SMAD signaling and inactivating CAFs by reducing collagen	TNBC	[24]
	Liposome	Silybin	Inactivation of CAFs by inhibiting NF-κB activation induces TME remodeling	Breast cancer	[25]
	Liposome	Salvianolic acid B	Inhibiting TGF-β1/SMAD signaling and CAF activation, decreasing collagen deposition, alleviating the fibrotic environment, increasing the infiltration of CD8^+^ and CD4^+^ T cells into tumors, and increasing levels of Th1 cytokines while decreasing levels of Th2 cytokines	Breast cancer	[114]
Reprogramming/normalizing CAFs	Lipid NP	JQ1	JQ1 normalizes CAFs by reducing the expression of genes associated with aberrant activation in CAFs, remodeling the ECM, and promoting immune cell infiltration	Pancreatic cancer	[133]
	Extracellular vesicles	Calcipotriol	Reprogramming CAFs into normal fibroblasts reduced the tumor ECM, effectively regulating T-cell infiltration into the tumor	Liver cancer	[207]
	Mesoporous silica NP	Etinoic acid	Reversing CAF activation effectively overcame the physical barrier formed by deposited collagen and abnormal blood vessels, promoting the infiltration of immunostimulatory cells	Liver cancer	[209]
	Liposome	Ginsenoside Rg3	Reprograming activated CAFs into resting CAFs, attenuating the dense stromal barrier by inhibiting TGF-β secretion by tumor cells, modulating TGF-β/SMAD signaling, and reversing immunosuppression	Breast cancer	[209, 210]
Depletion/killing CAFs	Nanogel	S-Nitrosoglutathione	Activated CAFs are sensitive to NO, reducing TGF-β secretion by killing CAFs reduces the non-differentiation of monocytes recruited at the tumor site into M2-type macrophages	Breast cancer	[211]
	Polymer NP	Nintedanib/ABT-263	Reducing the secretion of immunosuppressive factors, clearing and aging CAFs, and reshaping the tumor immunosuppressive microenvironment	Breast cancer	[198]
	Cu(II) MOFs	Blebbistatin	Inducing apoptosis of CAFs by mediating the photogeneration of •OH to inhibit ECM production, synergize oxidative stress in tumors, and activate antitumor immune responses	Breast cancer	[125]
	Au NP	DOX	Reduced CAFs by triggering the photothermal effect, remodeling the TME and increasing the number of NK cells in the tumor	Breast cancer	[124]
	Polymeric micelles	Tranilast/DOX	Optimises TME mechanoregulation and tumor perfusion by reducing fibrotic conductance in CAFs. Durable long-term antitumor response and immune memory in combination with chemotherapeutic agents	Breast cancer	[108]
	FeCo-ZIF	Telmisartan	Enhances drug penetration into tumors and infiltration of cytotoxic T lymphocytes by specifically killing CAFs, destroying ECM, and delaying CXCL12 secretion	Breast cancer	[128]
	Lipid NP	Paclitaxel/PFK15	Reduced lactate production of lactate and reduced production of immunosuppressive factors by blocking the metabolic support of CAFs to cancer cells	Breast cancer	[129]

### Inhibition of CAF activation

CAF activation is influenced by multiple factors and signaling pathways in the TME, and these activated CAFs play an important role in tumorigenesis and progression. Several methods can usually be used to inhibit CAF activation, such as TGF-β receptor inhibitors (e.g.,Galunisertib) [9], FAP inhibitors(e.g.,Talabostat mesylate) [110], Fibroblast adhesion patch kinase (FAK) inhibitors(e.g.,IN10018) [212], Angiotensin II Receptor Antagonists (ARBs) inhibitors(e.g., Valsartan、Losartan) [110, 138], tyrosine kinase (TK) inhibitors(e.g.,Sunitinib、Nintedanib)[213, 214], NF-κB inhibitors(e.g.,Silybin) [121, 140], and TGF-β/SMAD signaling pathway inhibitors(e.g.,Baicalein、Salvianolic acid B) [24]. In addition, CAF activation was effectively inhibited by reversing EMT [127].

**Fig. 5:**
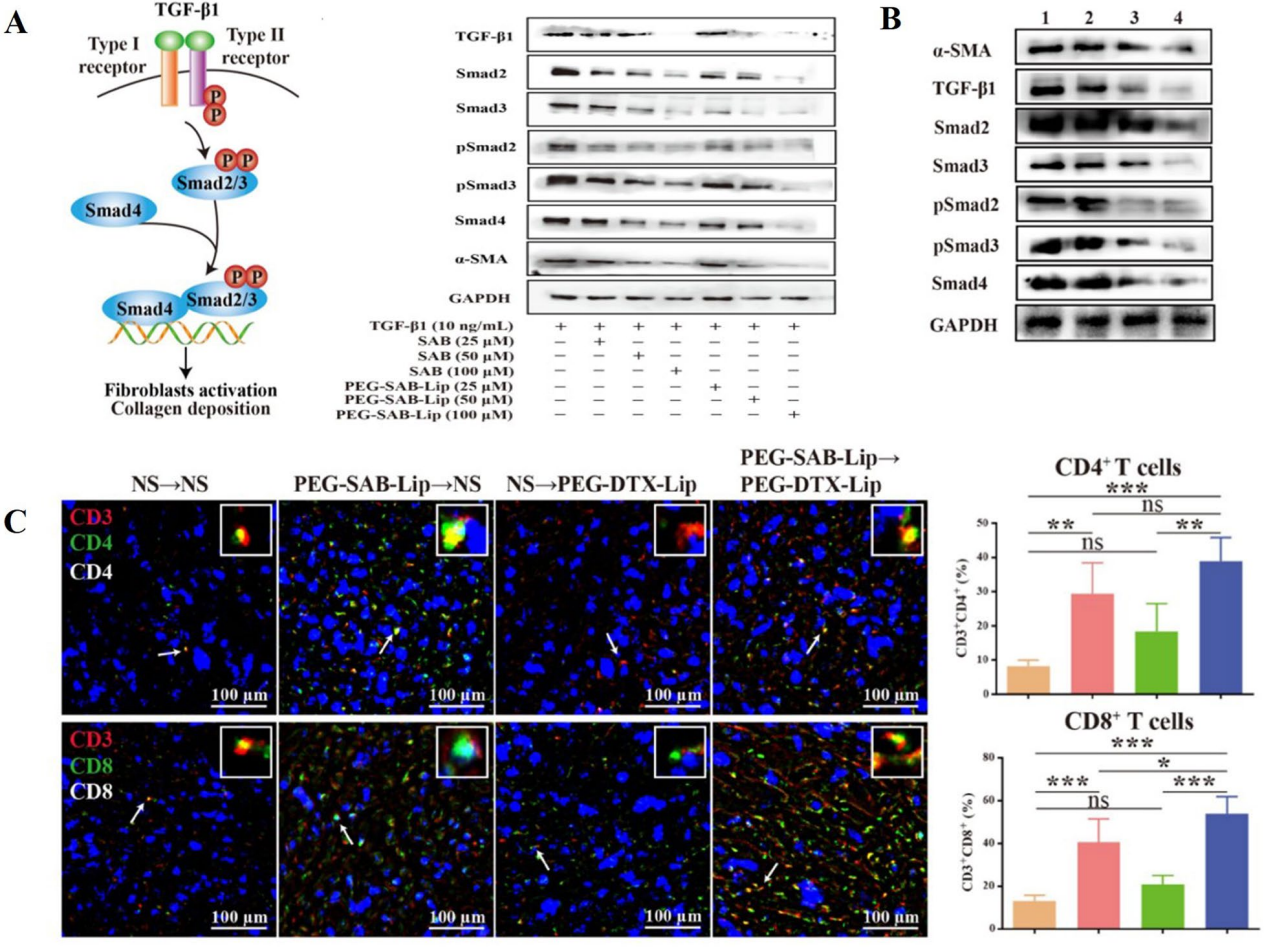
**A** Western blot bands and semi-quantitative analysis of the effects of SAB and PEG-SAB-Lip on the TGF-β1/Smad signaling in TGF-β1-activated NIH3T3 cells. **B** Western blot analysis of the levels of tumor proteins after different treatments. 1: NS, 2: PEG-B-Lip, 3: SAB, 4: PEG-SAB-Lip. **C** Immunofluorescence staining and the corresponding quantitative analysis of CD4^+^T and CD8^+^T immune functional cells in the TME under the combined therapy of PEG-SAB-Lip and PEG-DTX-Lip. Adapted with permission from [114] (copyright © 2023 American Chemical Society.)

These inhibitory strategies often rely on the encapsulation of the drugs in nanomaterials for improved delivery and efficacy. For instance, Chen et al. developed PEGylated liposomes loaded with SAB (PEG-SAB-Lip). These liposomes remodel the TME by inhibiting the TGF-β1/Smad signaling pathway in activated fibroblasts [114]. Furthermore, the impact of PEG-SAB-Lip on key proteins such as α-SMA, TGF-β1, Smad2, Smad3, and pSmad2 was found to be significantly stronger than that of the SAB group (Fig. 5A, B). In addition to targeting CAFs, PEG-SAB-Lip also enhances the anti-tumor effects of docetaxel and significantly increases the expression of CD4^+^ and CD8^+^T cells (Fig. 5C). In another study, Liang et al. encapsulated Valsartan and doxorubicin (DOX) in polymer micelles. Valsartan inhibits CAF activity, while DOX penetrates deep into tumor cells, inducing cellular senescence and recruiting effector immune cells [138].

### Reprogramming of CAFs

**Fig. 6:**
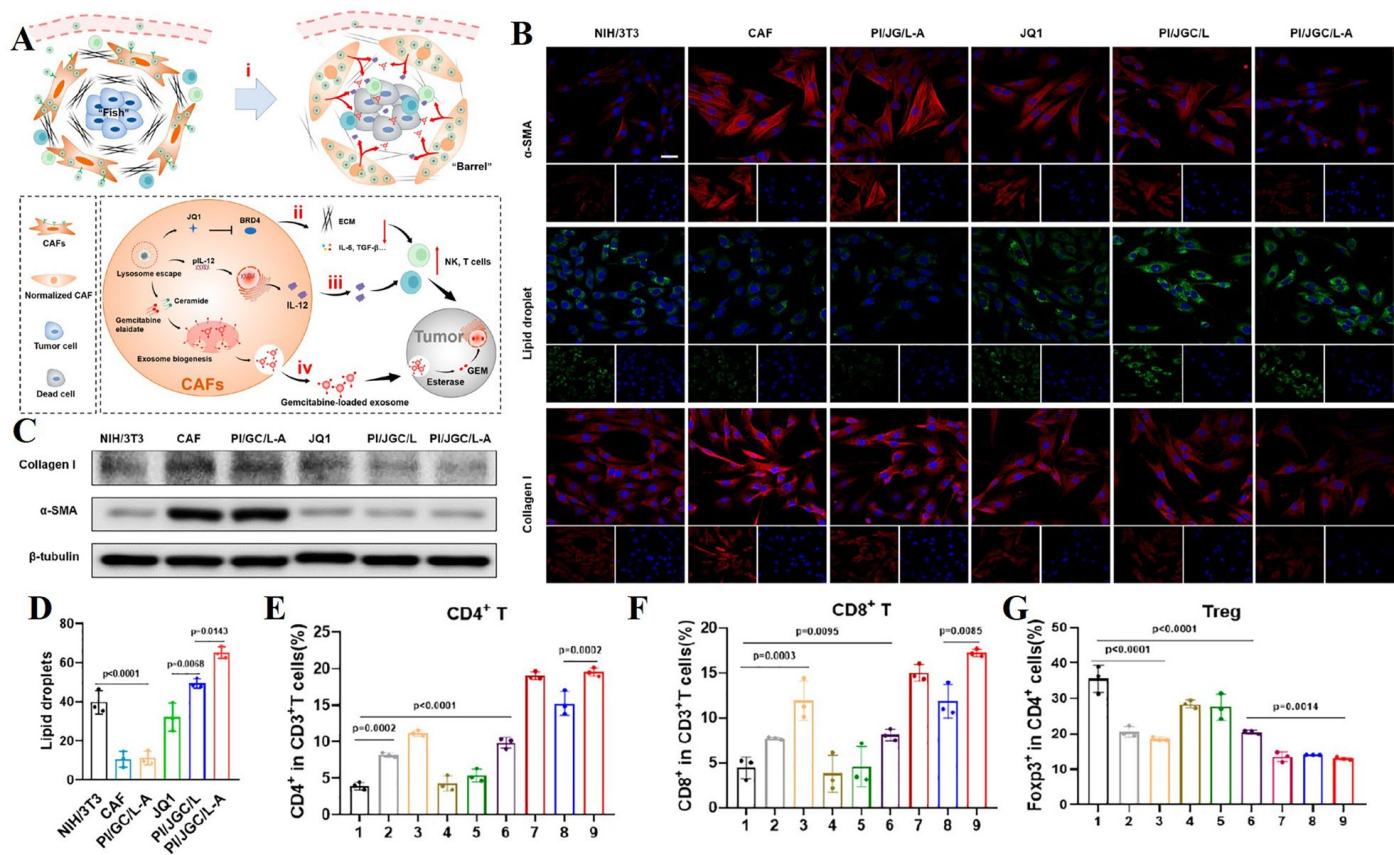
**A** Mechanism of Action of Lipid Nanoparticles (**B**) Immunofluorescence (IF) Staining of α-SMA and collagen I, alongside BODIPY 493/503 staining of lipid droplets in CAFs after 48 h of culture with the formulation containing JQ1. Scale bar = 40 μm. **C** Western Blot Analysis of α-SMA and collagen I in CAF lysates following 48-h treatment with the formulation containing JQ1. **D** Quantification of Lipid Droplets: The average number of lipid droplets per 10 CAFs. **E**–**G** Flow Cytometry (FCM) Analysis of CD4^+^ T cells, CD8^+^ T cells, and Tregs within the TME. Adapted with permission from [133] (copyright © 2023 American Chemical Society.)

Currently, one of the recommended strategies to inhibit tumor development is to modify CAFs to restore their normal functions, transforming them from “tumor-promoting” to “tumor-suppressing” cells. To achieve this, various molecules have been integrated into nano-delivery systems to regulate CAFs and enhance the efficacy of cancer immunotherapy. Notable examples include Calcipotriol [207], Retinoic Acid (RA) [209], All trans-retinoic acid (ATRA), the bromodomain-containing protein 4 (BRD4) inhibitor (e.g., JQ1) [133, 197], and melittin, the principal component of Apis mellifera venom [215]. For instance, Yuan et al. encapsulated JQ1, interleukin-12 (IL-12), and Elaidate in lipid nanoparticles, which exerted combined chemo-immunological effects (Fig. 6A). These lipid nanoparticles significantly reduced the expression of α-SMA and collagen I (Fig. 6B, C), while promoting the generation of lipid droplets (Fig. 6D), thereby facilitating CAF normalization. By decreasing ECM deposition, the lipid nanoparticles also enhanced immune cell infiltration (Fig. 6E–G) [133]. In another study, Zhang et al. created a biomimetic delivery system by coating liposomes with CAF membranes and co-encapsulating Pirfenidone and JQ1. This liposomal system specifically targeted CAFs, decreasing their activity and collagen production [197].

In addition, promoting the normalization of CAFs' functions has shown potential in improving the treatment of desmoplastic tumors. These tumors are characterized by intratumoral stiffness and mechanical forces that compress tumor blood vessels, reducing perfusion and hindering the infiltration of both drugs and immune cells [105]. Strategies to address this issue include directly targeting the tumor stroma or modulating CAF activity. Common therapeutic agents used for this purpose include antifibrotic drugs, antihypertensive agents, and corticosteroids. For example, Myrofora et al. encapsulated Tranilast in polymer micelles to control fibrotic signaling in CAFs. This approach modulated the mechanical properties of the TME, improved tumor perfusion, and significantly enhanced the uniform accumulation of cytotoxic nanodrugs, leading to stronger anti-tumor effects. Furthermore, these polymer micelles also promoted T-cell infiltration and induced long-term immune memory [108]. Similarly, Zhu et al. encapsulated Sunitinib in lipid-calcium sulfate nanoparticles and combined it with vaccination immunotherapy to improve the immunosuppressive environment of fibrotic, collagen-rich desmoplastic melanoma [214]. Additionally, remodeling the stromal environment can be achieved by targeting key downstream mediators of pro-fibrotic pathways, such as reactive oxygen species (ROS). Xu et al. developed a novel puerarin nanoemulsion that downregulated intratumoral ROS levels, thereby inactivating CAFs, reducing collagen deposition, and alleviating desmoplasia [133].

### Depletion of CAFs

Enhancing cancer immunotherapy can also be achieved by targeting and depleting CAFs. Small interfering RNA (siRNA) technology can be used to affect the interaction between CAFs and cancer cells by reducing the expression of specific target genes such as hepatocyte growth factor (HGF). The combined use of siRNA and chemotherapeutic agents like doxorubicin (DOX) has been shown to effectively deplete CAFs and reduce their secretion of HGF, thereby inhibiting their tumor-promoting role [216, 217]. Similarly, applying a TGF-β siRNA (siTGF-β) to tumor cells and CAFs significantly reduced their expression of TGF-β, helping to normalize the vascular system of the tumors, inhibiting EMT, and increasing the infiltration of immune cells [149]. Moreover, activated CAFs are sensitive to nitric oxide (NO), a property that has also been utilized to improve CAF-mediated tumor immunosuppression [211]. However, excessive depletion or killing of CAFs could potentially disrupt the functions of normal tissues. Therefore, researchers are working on refining strategies to ensure both safety and efficacy. For instance, Akai et al. targeted FAP and developed a near-infrared photoimmunotherapy approach, which successfully and selectively depleted CAFs without affecting normal cells. This strategy effectively eliminated the immunosuppressive CAFs and remodeled the local tumor immune microenvironment [217].

### Targeted CAFs therapy in combination with adjunctive treatment modalities

#### Combination with photothermal therapy (PTT)

**Fig. 7:**
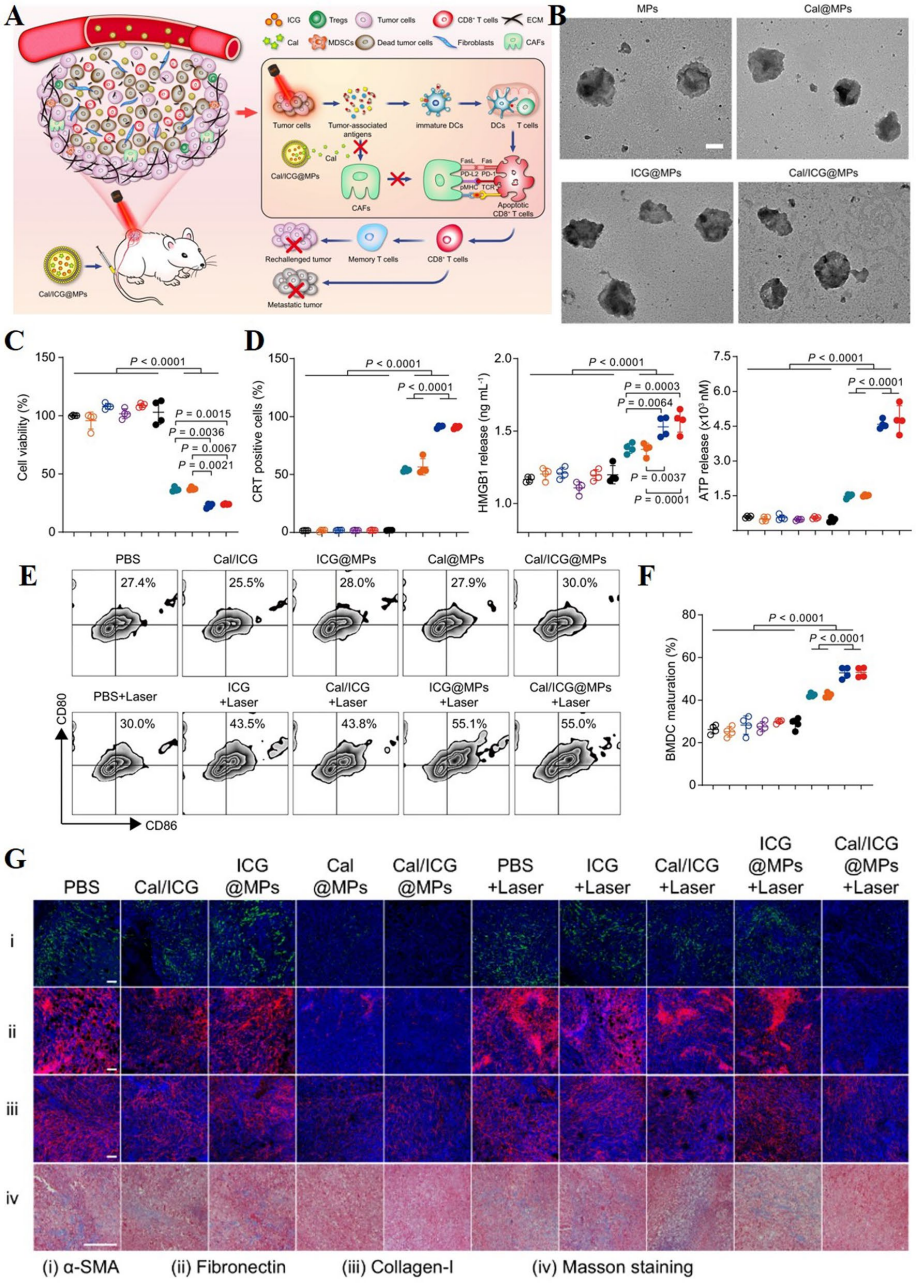
**A** Mechanism diagram of Cal/ICG@MPs. **B** TME images of MPs, Cal@MPs, ICG@MPs, and Cal/ICG@MPs. **C** Cell viability under different treatment groups with or without 808 nm laser irradiation. **D** Analysis of CRT, HMGB1, and ATP levels. **E** Flow cytometric analysis of BMDCs. **F** Semi-quantitative analysis. **G** Immunofluorescence staining for α-SMA, fibronectin, and collagen-I, along with Masson’s trichrome staining. Adapted with permission from [207] (copyright © 2022 Springer Nature Limited)

Photothermal therapy (PTT) treats tumors by irradiating targeted photothermal materials or photosensitizers with near-infrared (NIR) light, inducing localized thermal effects that can effectively kill tumor cells and modulate the immune response by altering the tumor microenvironment [218]. However, the presence of CAFs significantly hinders the efficacy of PTT, as CAFs secrete ECM components, such as collagen and hyaluronic acid, which create physical barriers that limit therapeutic effectiveness. Existing research has shown that PTT can alleviate tumor hypoxia and enhance the efficacy of PDT [219]. A range of photosensitizers, such as indocyanine green (ICG), and photothermal materials, including PDA and gold nanomaterials, have been successfully targeted and delivered to tumor sites. Upon external light irradiation, these materials generate localized hyperthermia, directly heating the tumor tissue [124, 201, 206, 219]. Wang et al. developed polydopamine (PDA) particles coated with CAF membranes and tumor cell membranes. Through the PDA-mediated photothermal effect, the tumor stroma was relaxed, thereby disrupting the physical barriers formed by the ECM and facilitating deeper drug penetration and targeting of tumor cells [158]. Similarly, Li et al. encapsulated calcipotriol and indocyanine green (ICG) in microparticles (MPs) to create Cal/ICG@MPs. Upon 808 nm laser irradiation, this formulation induced a potent photothermal effect that triggered immunogenic cell death (ICD) in tumor cells (Fig. 7A, B) [207]. Notably, the percentage of calreticulin (CRT)-positive cells in the ICG@MPs and Cal/ICG@MPs treatment groups was significantly higher than in the free ICG and Cal/ICG groups, with enhanced secretion of HMGB1 and release of M (Fig. 7C, D). Moreover, the Cal/ICG@MPs treatment promoted the maturation of DCs and the activation of CD8^+^ T cells (Fig. 7E, F). Importantly, it also increased the infiltration of CD8^+^ T cells into the deeper regions of the tumor by modulating CAF activity (Fig. 7G).

#### Combination with photodynamic therapy (PDT)

**Fig. 8:**
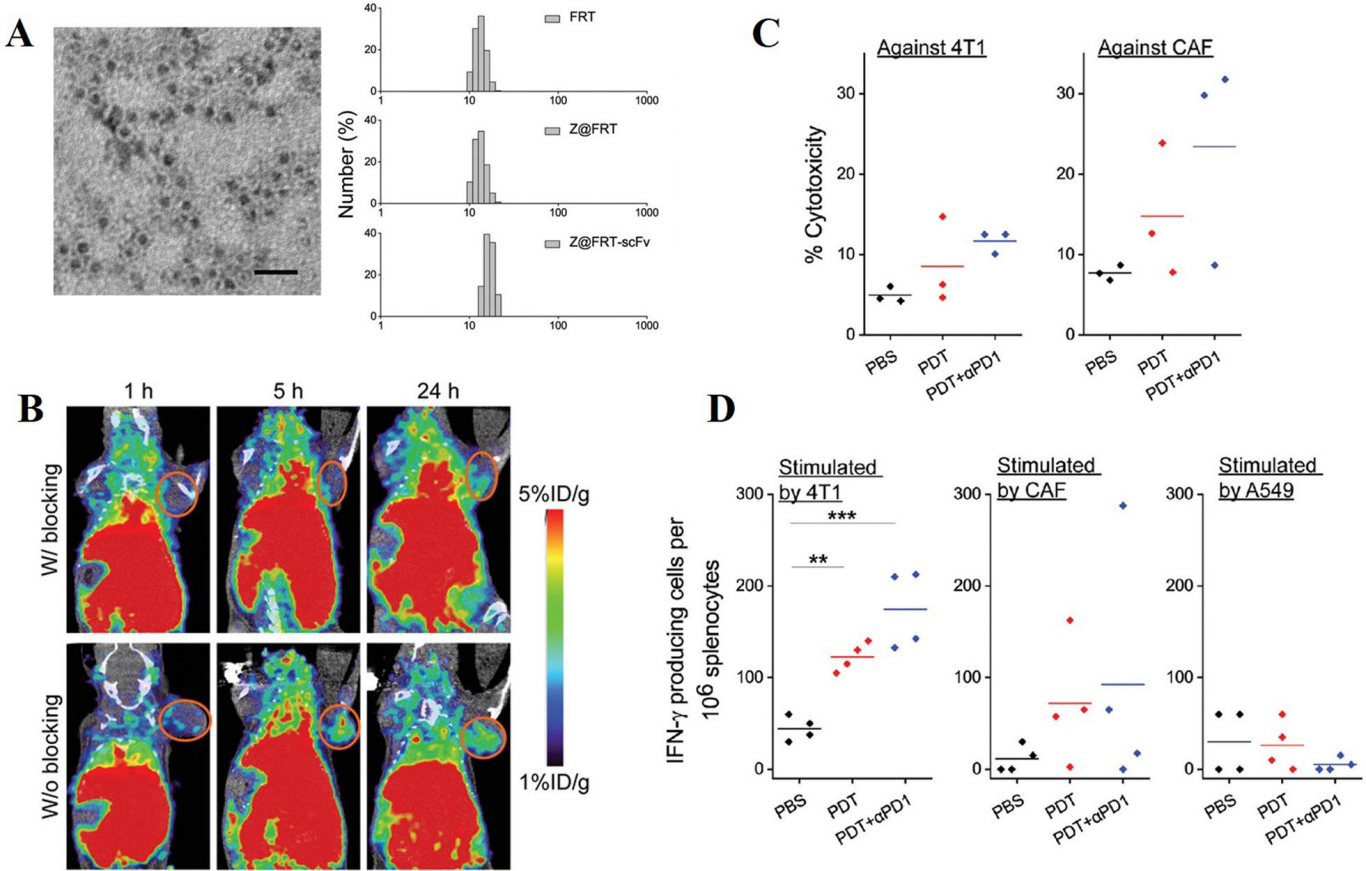
**A** TEM images of ferritin (FRT) and DLS analysis of αFAP-FRT, Z@FRT, and αFAP-Z@FRT. **B** PET imaging of 64Cu-labeled αFAP-FRT injected into 4T1-bearing mice, with images taken at 1, 5, and 24 h post intravenous injection. **C** Cell-specific cytotoxicity analysis for different groups. **D** Enzyme-linked immunospot (ELISpot) analysis for different groups.Adapted with permission from [222] (copyright © 2020 Wiley–VCH GmbH.)

Photodynamic therapy (PDT) utilizes photosensitizers to generate reactive oxygen species (ROS) under specific light irradiation, which can not only induce tumor cell death but also enhance tumor immunotherapy through mechanisms such as the release of tumor antigens and immune activation [220]. The ROS produced during PDT can also modulate the phenotypes and functions of cancer-associated fibroblasts (CAFs), thereby influencing their role in promoting tumor progression. PDT's therapeutic efficacy can be augmented either by optimizing photosensitizer use or by leveraging the intrinsic properties of nanomaterials to increase intracellular ROS levels. Tian et al. developed an alginate (ALG)-based hydrogel drug delivery platform (ALG@TPN), which incorporates TBP-2, Pt (0), and Nintedanib. The combined effect of PDT and chemotherapy activates the cGAS/STING pathway, leading to CAF inhibition and remodeling of the tumor immunosuppressive microenvironment [221]. Moreover, Zhou et al. engineered ferritin nanoparticles (αFAP-Z@FRTs) that conjugate a photosensitizer (ZnF^+^16Pc) with a fibroblast activation protein (FAP)-specific single-chain antibody (Fig. 8A) [222]. This design enables targeted delivery of PDT to CAFs, significantly enhancing accumulation at the tumor site (Fig. 8B). In both the PDT and combination therapy groups, the number of damaged CAFs surpasses that of 4T1 cancer cells, suggesting that the treatment may trigger an immune response against CAFs (Fig. 8C). Furthermore, the frequencies of effector T cells targeting both 4T1 tumor cells and CAFs are markedly elevated, indicating that these therapeutic strategies can activate the immune system, leading to an increased generation of effector T cells. This, in turn, may enhance the immune response against both cancer cells and CAFs (Fig. 8D).

#### Combination with immune checkpoint inhibition therapy

Direct interactions between cancer cells and CAFs have been shown to promote the upregulation of programmed death-ligand 1 (PD-L1) expression. This upregulation can be targeted by anti-PD-L1 antibodies, which not only enhance tumor cell death but also induce apoptosis in CAFs [224]. Programmed death 1 (PD-1) inhibitors, a class of immune checkpoint inhibitors (ICIs), work by reactivating the anti-tumor activity of TILs. They achieve this by blocking the PD-1/PD-L1 interaction, which suppresses TIL function under normal conditions. For instance, Guo et al. developed a system to encapsulate Oxymatrine and Astragaloside IV in MOFs. Oxymatrine (Om) was employed to inhibit CAF activation, thereby increasing the presence of TILs in the tumor microenvironment. Meanwhile, Astragaloside IV (As) enhanced the anti-tumor efficacy of TILs by improving their mitochondrial function. When combined with α-PD-1 antibodies, this therapeutic system significantly improved the anti-hepatocellular carcinoma(HCC) response, resulting in a tumor inhibition rate of 84.15% and extending the survival time of tumor-bearing mice [127].

**Fig. 9:**
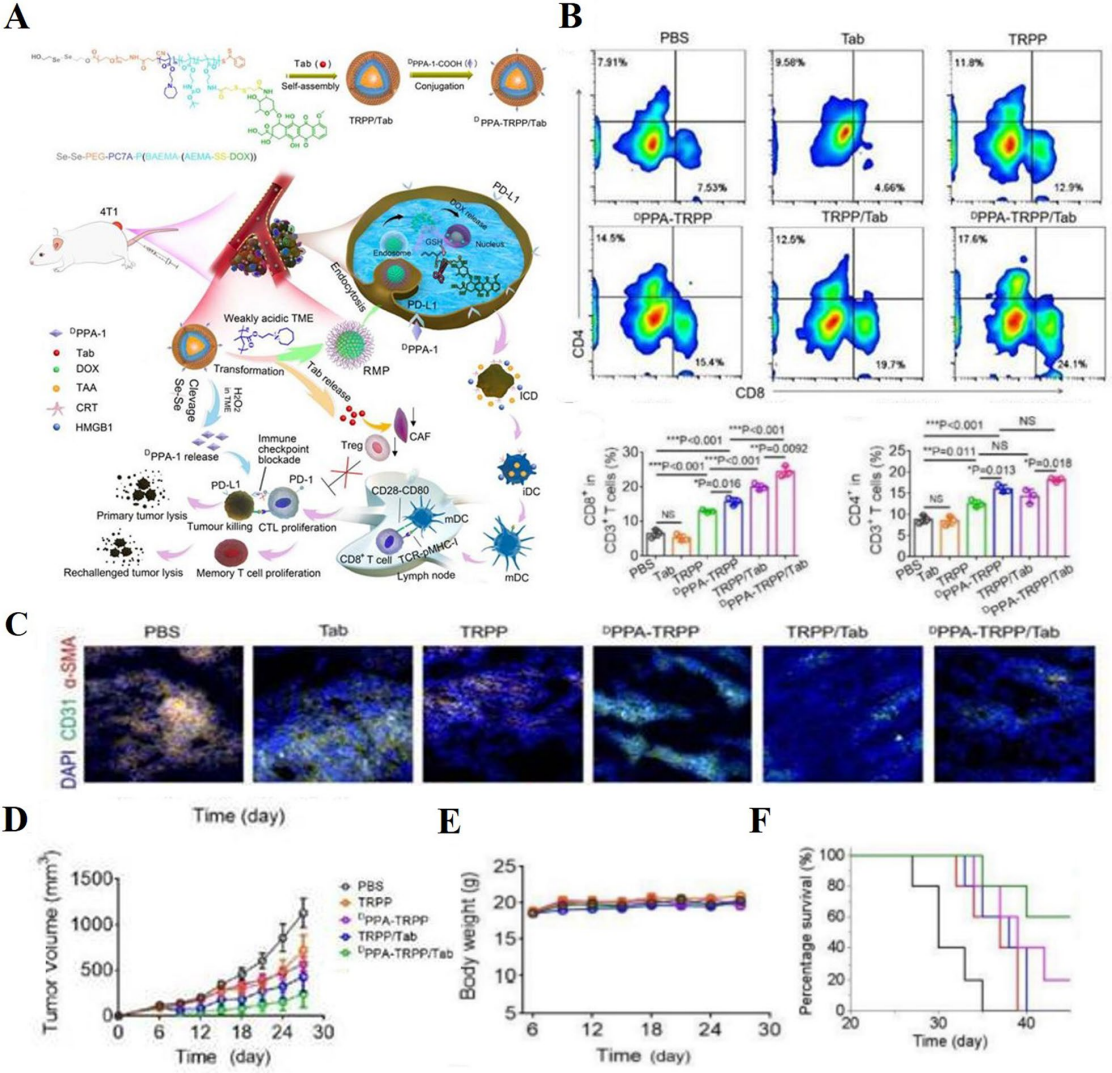
**A** Preparation and mechanism of action of PPA-TRPP/Tab. **B** Quantitative analysis of representative CD8^+^、CD4^+^T cell percentages after treatment in different groups and measurement of tumor tissues after different treatments using flow cytometry. **C** The expression of α—SMA after treatment in different groups is used to characterize CAFs (**D**) The average tumor growth curve (**E**) weight curve, **F** and survival curve. Adapted with permission from [110] (copyright ©2023 Ivyspring International Publisher.)

Furthermore, Yang et al. developed an innovative polymeric vesicle-based prodrug nanoplatform (TRPP/Tab) designed to simultaneously target CAFs and induce ICD, thereby enhancing the efficacy of cancer immunotherapy. This nanoplatform encapsulated three active compounds—DPPA-1, doxorubicin (DOX), and Talabostat mesylate—within polymeric micelles (Fig. 9A). In vivo, Talabostat mesylate effectively inhibited CAFs and reduced the accumulation of excessive reactive oxygen species (ROS), while DPPA-1 blocked immune checkpoints and doxorubicin (DOX) triggered ICD. Experimental results demonstrated that the DPPA-TRPP/Tab nanoplatform not only increased tumor accumulation but also suppressed CAF formation (Fig. 9C) and enhanced the activation of CD4^+^ and CD8^+^ T cells (Fig. 9B). As a result, this combination therapy achieved a 60% complete tumor regression rate and induced a durable immune memory response in the mouse model (Fig. 9D, E) [110].

#### Combination with CAR-T cell therapy

CAR-T cell therapy has the characteristics of self-expansion and higher sensitivity to low antigen expression, thus having the potential to become a promising alternative strategy for targeting fibroblast activation protein (FAP) tumor stromal cells. For example, Das et al. designed chimeric antigen receptor (CAR) T cells targeting fibroblast activation protein (FAP) through the transcription activator-like effector nuclease (TALEN) gene editing platform, which can effectively deplete cancer-associated fibroblasts CAFs in the tumor microenvironment TME and reduce the physical barriers of the stroma, thereby facilitating the infiltration of T cells [225].

#### Combination with microorganisms

Bacteria and their components have demonstrated significant potential as immunomodulators [226]. For instance, Bacillus Calmette-Guérin (BCG) has been established as the first successful immunotherapy. Research has shown that Trehalose Dimycolate (TDM), the most abundant hydrophobic glycolipid on the cell wall of BCG, possesses notable anti-tumor properties along with various immunostimulatory activities. Li et al. have explored the use of mesoporous silica nanoparticles loaded with TDM to enhance tumor fibrosis [227].

Moreover, engineered bacteria have emerged as powerful tools for augmenting cancer immunotherapy. Zhang et al. designed a genetically engineered bacterium capable of secreting nattokinase (NKase) [228]. This bacterium was developed by attenuating the Salmonella typhimurium strain VNP20009 and incorporating a gene encoding nattokinase into its plasmid via genetic engineering. The engineered bacterium can reprogram CAFs from an activated to a quiescent state by secreting substantial amounts of NKase, which degrades fibronectin within the tumor microenvironment. This modification alters the ECM and results in a reduction in tumor tissue stiffness, solid stress, and interstitial fluid pressure, thereby enhancing the efficacy of subsequent radiotherapy and immunotherapy.

## Future perspectives

CAFs are critical drivers of tumor progression, shaping the TME and influencing multiple aspects of cancer biology. Beyond supporting tumor growth and metastasis, CAFs contribute to immune evasion and the establishment of a pro-inflammatory milieu. They secrete a variety of cytokines, growth factors, and ECM components that promote tumor cell proliferation, survival, and migration. CAFs also facilitate ECM remodeling, enabling tumor cell invasion and metastasis. Recent insights into CAFs' role in EMT and the activation of key signaling pathways, such as TGF-β and Wnt/β-catenin, have deepened our understanding of the molecular mechanisms driving tumor progression. Moreover, CAF-immune cell interactions further complicate tumor immunology, modulating immune surveillance and influencing therapeutic responses. Looking ahead, targeting CAFs represents a promising therapeutic strategy for cancer treatment. However, the heterogeneity of CAFs, their dynamic behavior, and the complexity of their interactions with the tumor microenvironment present significant challenges for the development of effective therapies. Future research should focus on unraveling the precise molecular mechanisms underlying CAF function, as well as identifying specific biomarkers that can be used to selectively target CAFs without disrupting normal tissue homeostasis. Additionally, it is necessary to optimize these strategies for clinical application, addressing challenges related to specificity, safety, and tumor heterogeneity. The integration of CAF-targeted approaches into personalized cancer treatment regimens will likely be an important step toward achieving more effective and durable therapeutic responses. Thus, a deeper understanding of CAF biology will be essential for the development of novel, targeted therapies aimed at improving patient outcomes in cancer.

## Conclusions

This review provides an overview of recent advancements in CAF-targeted therapies and explores emerging techniques for the molecular targeting of CAFs. Despite being a predominant cell type in the tumor microenvironment, CAFs present a significant challenge for precise therapeutic targeting due to their inherent heterogeneity. Overcoming these challenges is essential to translating research findings from the laboratory to clinical applications. The origins of CAFs across different cancer types remain unclear, as does the full understanding of their subtypes and functional diversity. Additionally, minimizing off-target and systemic effects continues to be a major hurdle. Furthermore, combining CAF-targeted immunotherapies with existing therapeutic strategies holds great promise and is an active area of investigation. These approaches not only deepen our understanding of CAF biology but also have the potential to enhance the precision of cancer therapies and improve patient outcomes.

## Data Availability

No datasets were generated or analysed during the current study.

## Abbreviations

IL1R1/IL-1R: The IL-1/IL-1 receptor type 1
PKM/PKM2: Pyruvate kinase M1/2 (PKM/PKM2)
(NTRK2/TRKB)/NFE2: The neurotrophic receptor tyrosine kinase 2
ACTA2/α-SMA: Actin alpha 2, smooth muscle
ADORA2A: Adenosine A2A receptor
AKT: Protein kinase B
ALB: Albumin
ApCAFs: Antigen-presenting CAFs
BCG: Bacillus Calmette-Guérin
BDNF: Brain-derived neurotrophic factor
BP: Black phosphorus
BSA: Bovine serum albumin
CaCO_3_: Calcium carbonate
CAFs: Cancer-associated fibroblasts
CELF2: The CUGBP Elav-like family member 2
circFARP1: Pleckstrin domain protein 1
circZFR: The zinc finger RNA binding protein
CMC: Critical micelle concentration
CPG: Cytosine-phosphate-guanine
CRC: Colorectal cancer
CRMP2: Collapsin response-mediated protein 2
CSF2/GM-CSF: Colony-stimulating factor 2
CTLs: Cytotoxic T lymphocytes
CXCL2: C-X-C motif chemokine ligand 2
CXCL5: C-X-C motif chemokine ligand 5
CXCL9: C-X-C motif chemokine ligand 9
CXCL10: C-X-C motif chemokine ligand 10
CXCL12: C-X-C motif chemokine ligand 12
CXCL16: C-X-C motif chemokine ligand 16
CXCR4: C-X-C motif chemokine receptor 4
DAS: Dasatinib
DCs: Dendritic cells
DPPC: Dipalmitoylphosphatidylcholine
DSPE-PEG: 1, 2-Distearoyl-sn-glycero-3-phosphoethanolamine-N-PEG
ECM: Extracellular matrix
EGF: Epidermal growth factor
EMT: Epithelial-mesenchymal transition
EVs: Extracellular vesicles
FAK: Fibroblast adhesion patch kinase
FAP/FAP-α: Fibroblast activation protein alpha
FGF2: Fibroblast growth factor 2
FGF7: Fibroblast growth factor 7
FOXF2: Forkhead box F2
Foxp3: Forkhead box P3
GPR30: G protein-coupled receptor 30
HA: Hyaluronic acid
HAS2: Hyaluronan synthase 2
HCC: Hepatocellular carcinoma
HES-FA: Hydroxyethyl starch-folate conjugates
HIF1A/HIF-1α: Hypoxia-inducible factor
HPSE: Heparanase
iCAFs: Inflammatory CAFs
ICD: Immune cell death
IGFBP7: Insulin-like growth factor-binding protein 7
JAKs: Janus kinases
MAPK: Mitogen-activated protein kinase
MCT1: Monocarboxylate transporter1
MDSC: Mononuclear myeloid-derived suppressor cell
MMP: Matrix metalloproteinase
MOF: Metal–organic framework
myCAFs: Myofibroblasts
NF-κB: Nuclear factor kappa B
NKs: Natural killer cells
NO: Nitric oxide
NPs: Nanoparticles
OPN: Osteobridging protein
OV: Oncolytic virus
p62: Encoded by the SQSTM1 gene
PCL: Polycaprolactone
PDGF: Platelet-derived growth factor
PDGFRA/PDGFR-α: PDGF receptor alpha
PEG: Polyethylene glycol
PEG-PBLG: PEG-b-poly(benzyl-L-glutamate)
PF4/CXCL4: G protein-coupled receptor-mediated binding of platelet factor 4
PLA: Polylactic acid
PLA2G2A: Phospholipase A2 group IIA
PLGA: Biodegradable poly(lactic-co-glycolic) acid
POEGMA: Poly(oligo(ethylene glycol) methyl ether methacrylate)
PPBP/CXCL7: Pro-platelet basic protein
PSC: Pancreatic stellate cell
PVA: Polyvinyl alcohol
RA: Retinoic acid
ROS: Reactive oxygen species
SAB: Salvianolic acid B
SERPINE1/PAI1: Serpin family E member 1
sFRP4: Secreted frizzled-related protein
siRNA: Small interfering RNA
SOX4: SRY-box transcription factor 4
STAT3: Signal transducer and activator of transcription 3
TAMs: Tumor-associated macrophages
TDM: Trehalose Dimycolate
TGF-β: Transforming growth factor-beta
TPS: Trypsin-like protease
TILs: Tumor-infiltrating lymphocytes 4
TME: Tumor microenvironment
Tregs: Regulatory T cells
uPAR: The urokinase plasminogen activator receptor
VCAM1: Vascular cell adhesion molecule 1
VCAN: Versican
VEGFs: Vascular endothelial growth factors
WNT2: Wnt family member 2
WWOX: WW domain-containing oxidoreductase
