# Correction: Regulation of cancer‑associated fibroblasts for enhanced cancer immunotherapy using advanced functional nanomedicines: an updated review

**DOI:** 10.1186/s12951-025-03344-8

**Published:** 2025-04-04

**Authors:** Tingting Liao, Xiaoxiao Chen, Fengkai Qiu, Xinyu Zhang, Fazong Wu, Zhongwei Zhao, Ming Xu, Minjiang Chen, Jia-Wei Shen, Qiying Shen, Jiansong Ji

**Affiliations:** 1 School of Pharmacy, College of Pharmacy, Hangzhou Normal University, 2318 Yuhangtang Road, Hangzhou, 310015 Zhejiang China; 2 Zhejiang Key Laboratory of Imaging and Interventional Medicine, The Fifth Affiliated Hospital of Wenzhou Medical University, 289 Kuocang Road, Lishui, 323000 China; 3 Department of Radiology, Lishui Central Hospital, The Fifth Affiliated Hospital of Wenzhou Medical University, Lishui, 323000 China; 4 Cixi Biomedical Research Institute, Wenzhou Medical University, Ningbo, 315300 China; 5 Key Laboratory of Elemene Class Anti-Cancer Chinese Medicines, Engineering Laboratory of Development and Application of Traditional Chinese Medicines, Collaborative Innovation Center of Traditional Chinese Medicines of Zhejiang Province, Hangzhou Normal University, Hangzhou, 311121 China

**Correction: Journal of Nanobiotechnology (2025) 23:166**

10.1186/s12951-025-03217-0

In this article, under the sections ‘Modulation of tumor immunity’ ‘BP‑based nanomaterials’ and ‘Reprogramming of CAFs’, some of the texts incorrectly appeared in Chinese characters instead of English and should be removed.

The original article has been corrected.

